# Targeting CC chemokine ligand (CCL) 20 by miR-143-5p alleviate lead poisoning-induced renal fibrosis by regulating interstitial fibroblasts excessive proliferation and dysfunction

**DOI:** 10.1080/21655979.2022.2062106

**Published:** 2022-04-29

**Authors:** Lin Han, Yanfang Zou, Chen Yu

**Affiliations:** a Department of Nephrology, Yangpu Hospital, School of Medicine, Tongji University, Shanghai, China; b Department of Nephrology, Tongji Hospital, School of Medicine, Tongji University, Shanghai, China

**Keywords:** Renal fibrosis, lead contamination, CCL20, miR-143, renal *interstitial fibroblast*

## Abstract

Environmental lead contamination can cause chronic renal disease with a common clinical manifestation of renal fibrosis and constitutes a major global public health threat. Aberrant proliferation and extracellular matrix (ECM) accumulation in renal interstitial fibroblasts are key pathological causes of renal fibrosis. However, the mechanism underlying lead-induced kidney fibrosis remains unclear. The present study analyzed gene expression prolifes in lead acetate-treated primary mice renal interstitial fibroblasts and confirmed the aberrant expression of CC chemokine ligand (CCL) 20, one of the most obvious up-regulated genes. Analogously, lead acetate exposure dose-dependently increased CCL20 transcription, protein expression and release. Knockdown of CCL20 suppressed lead acetate-induced fibroblast proliferation, hydroxyproline contents, transforming growth factor-beta production and ECM-related protein (Collagen I and fibronectin) expression. Bioinformatics analysis predicted five top miRNAs targeting CCL20. Among them, miR-143-5p expression was dose-dependently decreased in lead acetate-treated fibroblasts. Mechanistically, miR-143-5p directly targeted CCL20. Elevation of miR-143-5p antagonized lead acetate-induced fibroblast proliferation, hydroxyproline and ECM-related protein expression, which were reversed by CCL20 overexpression. Additionally, CCL20 knockdown suppressed lead acetate-mediated Smad2/3 and AKT pathway activation. Notably, miR-143-5p overexpression attenuated the activation of the Smad2/3 and AKT pathway in lead acetate-exposed fibroblasts, which was counteracted by CCL20 elevation. miR-143-5p injection ameliorated renal fibrosis progression in mice *in vivo*. Thus, targeting CCL20 by miR-143-5p could alleviate renal fibrosis progression by regulating fibroblast proliferation and ECM deposition via the Smad2/3 and AKT signaling, providing a potential therapeutic target for environmental lead contamination-evoked fibrotic kidney disease.

## Graphical Abstract

**Figure:**
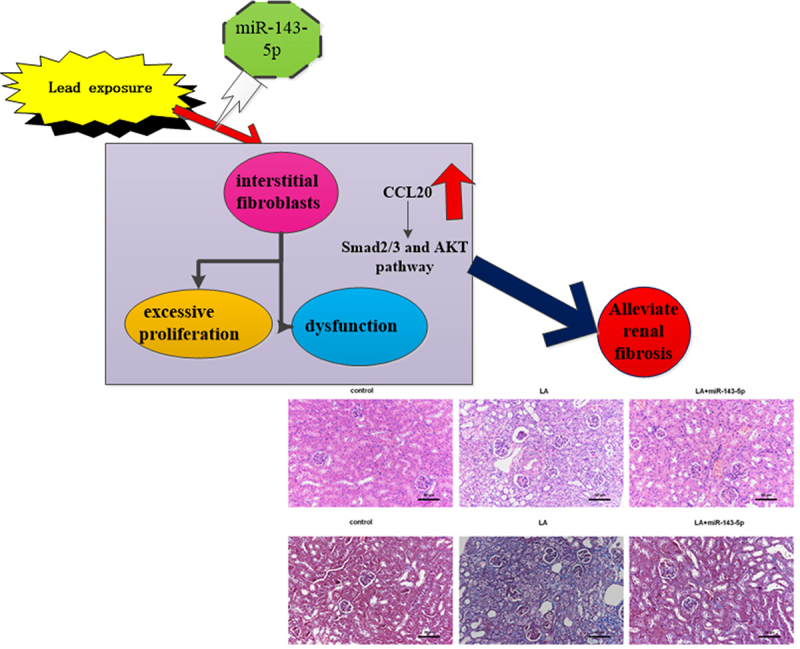

## Highlights

- Lead acetate (LA) increases CCL20 expression in mouse renal interstitial fibroblasts
- CCL20 loss alleviates LA-induced excessive proliferation, dysfunction of fibroblasts
- miR-143-5p antagonizes LA-induced dysfunction of fibroblasts by targeting CCL20
- miR-143 targets CCL20 to affect Smad2/3 and AKT signaling in LA-treated fibroblasts
- miR-143 attenuates renal fibrosis progression in LA-induced mice model

## Introduction

Chronic kidney disease (CKD) is a progressive condition that leads to abnormalities in kidney function and structure, and has become a major global public health concern with the prevalence increasing to 13% of the global population [1]. CKD prevalence usually results in high morbidity of end-stage renal disease and finally incurs an escalating economic burden worldwide [2,3]. Renal interstitial fibrosis represents a predominant pathological outcome in patients with CKD and is characterized by excessive proliferation of interstitial fibroblasts and accumulation of extracellular matrix (ECM) in interstitium [4,5]. Kidney fibrosis often causes renal dysfunction and ultimately end-stage renal failure, and its severity may predict the risk of long-term prognosis [6]. Consequently, it is of great significance to explore effective therapeutic strategy for renal fibrosis.

Except for common pathogenic risk factors, nephrotoxicity caused by chronic exposure to environmental toxins (for example, heavy-metals) has frequently been reported in epidemiological studies [7,8]. Among them, lead poisoning is a common heavy-metals contamination in environmental water, soil, and dust, and has been reported to induce various toxicities in the cardiovascular, liver, and renal systems [8,9]. Although lead contamination has declined over the last few decades, it remains a critical public health issue in high-income and lower- and middle-income countries [8,10]. Accumulating evidence supports the idea that lead exposure is associated with various chronic fibrotic progression, including renal fibrosis [7,9,11]. Decreased creatinine clearance and increased albuminuria have been observed among lead-exposed populations, which ultimately impair renal function and result in fibrosis in peritubular and interstitial lesions [4,11]. MicroRNAs (miRNAs) are small noncoding RNAs and can target various genes to participate in multiple physiological and pathological processes. Increasing evidence have implicated miRNAs in renal diseases, including renal fibrosis [12,13]. However, up to now, the underlying mechanisms involved in fibrosis associated with lead poisoning remains elusive.

In the current study, we analyzed gene expression profiles in lead acetate-treated primary renal interstitial fibroblasts using RNA sequencing (RNA-seq) and identified CC chemokine ligand (CCL) 20 as one of the most obvious up-regulated genes. Intriguingly, previous evidence has revealed the correlation between CCL20 and fibrotic progression in various diseases [14,15]. Therefore, we hypothesize that CCL20 may play the potential role in renal fibrosis. Thus, the current study investigated the role of CCL20 in lead acetate-induced proliferation and dysfunction of interstitial fibroblasts. Moreover, the miRNA that targets CCL20 was investigated during above processes. Additionally, the anti-fibrotic efficacy *in vivo* and underlying mechanism renal fibrosis were also elaborated. Intriguingly, the above results confirmed that targeting CCL20 by miR-143-5p alleviated lead acetate-induced renal fibrosis by regulating fibroblast proliferation and dysfunction, indicating a promising therapeutic approach for renal fibrosis.

## Materials and methods

### Animals and ethics statement

C57BL/6 mice (male, 8–10 weeks old) were purchased from SHANGHAI SLAC LABORATORY ANIMAL CO., LTD (Shanghai, China). Before experiments, all mice were raised in standard cages for at least 1 week to acclimatize to the environment and maintained at 22 ± 2°C on a 12 h light-dark cycle. All animals were fed standard chow diet and water *ad libitum*. During these process, all procedures were conducted according to the National Institutes of Health (NIH) Guide for the Care and Use of Laboratory Animals, and approved by the Institutional Animal Care and Use Committee of Yangpu Hospital, Tongji University School of Medicine (No.LL-2017-WSJ-002).

### Isolation of primary mice renal interstitial fibroblasts

Primary mouse renal interstitial fibroblasts were prepared as described previously [16]. Briefly, mice kidneys were collected and dissected into 1 mm^3^ pieces. Then, tissues were digested in 5 ml of DMEM medium supplemented with 10% fetal bovine serum (FBS), 1% penicillin/streptomycin and 2 mg/ml collagenase IV solution for 0.5 h at 37°C. After three repetitions, the prepared suspension was filtered through a 70-µm sterile filter. Following centrifugation at 1800 *g* for 5 min, cells were rinsed with PBS buffer and re-suspended in DMEM medium containing 10% FBS in an incubator with humidified atmosphere (5% CO_2_). Half of the medium was replaced every 3 days. After 80%–90% confluence, 0.25% trypsin and 0.02% EDTA were added for cell expansion. The adherent fibroblasts were identified by vimentin and CK19 staining, and analyzed by a confocal microscope. Cells between the second and sixth passages were used for the subsequent experiments.

### RNA-Seq analysis

TRIzol reagent (Invitrogen, Carlsbad, CA, USA) was used to extract total RNA from mice renal interstitial fibroblasts exposed to 1 μM lead acetate (LA; Sigma-Aldrich, St Louis, MO, USA). Then, a Ribo-ZeroTM rRNA Removal Kit was used to deplete the Ribosomal RNA. Subsequently, the cDNA libraries were constructed using Illumina NGS sequencing technologies by the Genergy Biotechnology (Shanghai, China). After that, all samples were sequenced using Illumina HiSeq2000. All raw data and different gene expression prolife were analyzed using R software by the Genergy Biotechnology (Shanghai, China). The threshold set for the statistical significance was log_2_|fold change (FC)| ≥ 1 and *P*-value < 0.05.

### Cell culture and lead acetate treatment

The isolated primary mouse renal interstitial fibroblasts were incubated with DMEM medium containing 10% fetal bovine serum, penicillin/streptomycin (100 U/ml) at 37°C and 5% CO2. For stimulation, cells were exposed to various doses of lead acetate (LA; Sigma, St Louis, MO, USA) (0–2 μM/L) for 24 h [17].

### Recombinant plasmid construction of CCL20

The prepared RNA was used as a template to synthesize the first-strand complementary DNA (cDNA) according to the instructions of the SuperScript II First Strand Synthesis System (Invitrogen). After PCR amplification, CCL20 cDNA was synthesized and digested with restriction enzymes to construct pcDNA-CCL20 plasmids by inserting CCL20 cDNA into pcDNA3.1(+) plasmids (Invitrogen) [18].

## Cell transfection

After seeding cells into a 6-well plate, cells were grown to 60–70% confluence. Then, cells were transfected with 40 nM small interfering (si)RNA control (si-NC), si-CCL20-1, si-CCL20-2 and si-CCL20-3, miR-143-5p mimics, miR-control (miR-con), pcDNA or pcDNA-CCL20 plasmids using the Lipofectamine® 2000 reagent (Invitrogen) [15,18]. All procedures were conducted according to manufacturer’s instructions. The siRNA sequences were obtained from JRDUN BiotechnologyCo.,Ltd (Shanghai, China). Approximately 48 h later, the effects on the expression of CCL20 and miR-143-5p were analyzed by qRT-PCR and western blotting.

## Real-time quantitative PCR (qRT-PCR)

Gene transcript levels were detected using the commercial SYBR Premix Ex TaqTM II Kit (Takara, Dalian, China) [17] according to the directions of manufacturers. The real-time PCR amplification reaction was denatured at 95 C for 10 min followed by a thermal cycling step of 40 cycles (95 C for 15 s; 60 C for 45 s). Primers were used as follows: CCL20 (F: 5'-CAACTCCTGGAGCTGAGAATG-3'; R: 5'-GAAGGAAGAGGCGTCTGTATG-3'); chemokine (C-C motif) receptor 6 (CCR6) (F: 5'-TGGAGCAGAATAGCAAGAAC-3'; R: 5'-GCAGAGGTGAAGCAATAATG-3'); transforming growth factor, beta 1 (TGF-β1) (F: 5'-TGGAAATCAACGGGATCAG-3'; R: 5'-ACAGAAGTTGGCATGGTAG-3'), fibronectin (FN) (F: 5'-TCTTTGAGTGGTCCTTTC-3'; R: 5'-ACCTGTGTTTCCCTATTG-3'), collagen, type I (Collagen I) (F: 5'-GCAACAGTCGCTTCACCTACAG-3'; R: 5'-CAATGTCCAAGGGAGCCACATC-3'). All reactions were performed on ABI Prism 7300 system and gene expression was assessed relative to internal controls, GAPDH or U6 using the 2^−ΔΔCt^ equation.

## Detection of cell proliferation

Cell proliferation was analyzed using a cell counting kit-8 (CCK-8) Kits [19] (Beyotime, Shanghai, China). Briefly, cells under various treatments were collected and then incubated with medium containing 10 μl of CCK-8 reagent (Beyotime) for 3 h. All procedures were conducted according to manufacturer’s instructions. Finally, the absorbance was detected at 450 nm using a spectrophotometer to assess cell proliferation.

## Establishment and administration of lead acetate-treated mice model

After acclimation to the laboratory conditions for 1 week, mice were randomly divided into three groups (n = 8 in each group). Group 1 served as control group that received the same dose of saline into abdomen. Group 2 was intraperitoneally injected with lead acetate (10 mg/kg) [20] for 4 weeks (LA groups). Group 3 served as LA and miR-143-5p groups wherein mice were given caudal vein injection of miR-143-5p mimics (1 OD/day) after LA injection for 2 consecutive weeks [19,21]. Then, plasma was then collected and stored at −70°C. Subsequently, mice were anesthetized and sacrificed by cervical dislocation without suffer anything to collect kidney tissues.

### ELISA assay

ELISA analysis was applied to determine the levels of TGF-β and CCL20 [22]. The supernatant of cells under various treatments and plasma was centrifuged and collected for subsequent detection. Then, the commercial ELISA kits of TGF-β and CCL20 were obtained from Abcam (Cambridge, UK, USA). A microplate reader was applied to determine the OD value at 450 nm to evaluate the contents of TGF-β and CCL20 in cellular supernatants and plasma of mice.

## Detection of hydroxyproline contents

After collection of plasma and cell supernatants, the contents of hydroxyproline were evaluated using a commercial Hydroxyproline Detection Kit (Nanjing Jiancheng Bioengineering Institute, Nanjing, China). Following the incubation with reaction buffer, samples were incubated at 60°C for 15 min. Then, supernatants were prepared after centrifuging for 10 min. The OD value at 550 nm was measured to evaluate hydroxyproline levels.

### Histological observations

The collected renal tissues were fixed in 4% formaldehyde and decalcified in 10% EDTA for 2 weeks. Then, samples were dehydrated and embedded in paraffin. After that, the specimens were cut into a serial 5-μm thick sections. The prepared renal sections were then stained with hematoxylin and eosin (H&E) and Masson’s trichrome (Beijing Leagene Biotechnology Co., Ltd., Beijing, China) to evaluate the degree of fibrosis [19].

### Western blotting analysis

Western blotting assay was performed to analyze protein expression as previously described [17]. Total protein from cells and kidney tissues was isolated using RIPA lysis buffer, and quantified using a BCA Protein Detection Kit (Beyotime). Subsequently, equal amounts of prepared protein were loaded into 12% SDS-PAGE and transferred to PVDF membranes. Then, bands were incubated with the primary antibodies against CCL20, CCR6, TGF-β1, FN, Collagen I, and p-Smad2/3 (Abcam), Smad2/3, AKT, and p-AKT (Cell Signaling Technology, Inc., Danvers, Massachusetts, USA) at 4°C overnight. Then, the membranes were incubated with horseradish peroxidase-conjugated secondary antibodies (Beyotime) at room temperature for 2 h, and then were covered with ECL Reagent (Beyotime). Subsequently, the immunoreactive bands were analyzed by a Gel DocTM XR imaging system (Bio-Rad Laboratories, Hercules, CA, USA) and quantified using ImageJ software (National Institutes of Health, Bethesda, MD, USA).

### Assay of miRNA-mRNA targets

For prediction of potential miRNAs targeting CCL20, the bioinformatics tool miRanda (http://www.microrna.org/microrna/home.do), Targetscan (http://www.targetscan.org/vert_71/) and miRWalk (http://mirwalk.umm.uni-heidelberg.de/) were used.

### Dual-Luciferase reporter assay

To verify the potential presence of complementary-binding sites between miR-143-5p and 3' untranslated region (UTR) of CCL20, a Dual-Luciferase reporter assay was applied [19]. In brief, a wild-type (WT) (pGL3-CCL20-wt) or mutant (Mut) (pGL3-CCL20-mut) luciferase reporter plasmid was constructed by inserting 3’UTR sequences of CCL20 into a luciferase reporter plasmid of pGL3 (Promega Corporation, Madison, WI, USA). Subsequently, the miR-NC, miR-143-5p mimics and pGL3-CCL20-wt or pGL3-CCL20-mut were co-transfected into cells for 48 h. The luciferase activity was then determined according to the instructions of a dual-luciferase reporter assay system (Promega) and normalized to *Renilla* luciferase fluorescence.

## Statistical analysis

Data are expressed as the mean ± standard deviation (SD) and were obtained from at least three parallel experiments. The statistical significance for three or more groups were analyzed by ANOVA with SNK comparison test using SPSS19.0 software. Differences in P < 0.05 were denoted as statistically significant.

## Results

### Noticeable upregulation of CCL20 is identified in mouse primary renal interstitial fibroblasts following exposure to lead acetate

**Figure 1.**
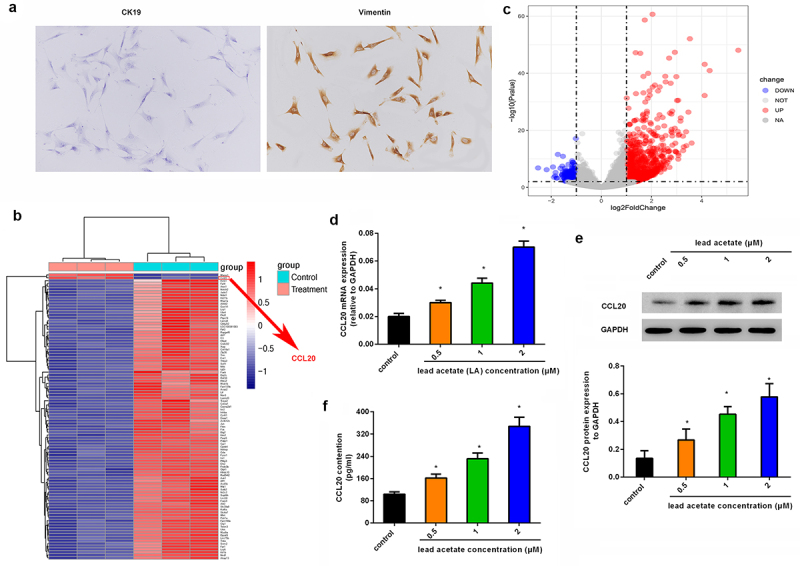
Aberrant expression of CCL20 is observed in lead acetate-stimulated mice primary renal interstitial fibroblasts. (a) Staining of renal interstitial fibroblasts with CK19 and Vimentin. (b) Heat map of gene expression in lead acetate-treated fibroblasts. The red shades represent high expression, and blue shades indicate low expression. (c) Volcano plots exhibited gene expression profiles. (d, e) Isolated renal fibroblasts were stimulated with the indicated doses of lead acetate for 24 h. Then, the mRNA (d) and protein expression (e) levels were analyzed by qRT-PCR and western blotting. (f) ELISA assay was performed to quantify the contents of CCL20 in supernatants from lead acetate-treated renal fibroblasts. *P < 0.05 vs. control groups.

Before experiments, we isolated primary renal interstitial fibroblasts from mice by staining them with CK19 (negative) and Vimentin (positive) (Figure 1a). To elucidate the underlying mechanism involved in lead-induced renal interstitial fibrosis, we characterized gene transcripts by analyzing gene expression profiles in lead acetate-treated primary renal interstitial fibroblasts (Figure 1b and 1c), and found that there were abundant aberrant gene expression. Herein, we mainly focused on the up-regulated genes; among them, CCL20 was one of the most prominently up-regulated genes (Figure 1b and 1c). Similarly, qRT-PCR assay revealed the dose-dependent increase in CCL20 mRNA levels in lead acetate-treated fibroblasts relative to control groups (Figure 1d). Concomitantly, lead acetate incubation also enhanced CCL20 protein expression (Figure 1e) and release from renal interstitial fibroblasts (figure 1f). These data indicate a potential role of CCL20 in lead-induced renal interstitial fibrosis.

### CCL20 knockdown alleviates lead acetate-induced excessive proliferation and dysfunction of renal interstitial fibroblasts

**Figure 2.**
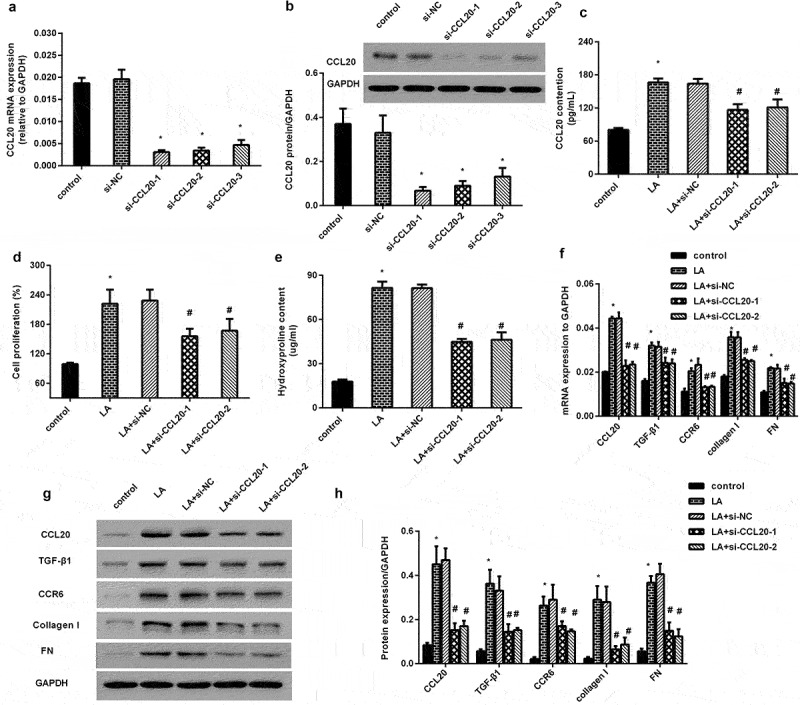
CCL20 knockdown inhibits lead acetate-evoked renal interstitial fibroblast proliferation and dysfunction. (a, b) Renal interstitial fibroblasts were transfected with si-CCL20-1, si-CCL20-2 and si-CCL20-3 for 48 h. Then, the mRNA levels of CCL20 (a) and protein expression (b) were detected by qRT-PCR and western. (c) Cells were transfected with si-CCL20-1, si-CCL20-2, and prior to lead acetate exposure. The contents of CCL20 in supernatants were analyzed by ELISA kits. (d) Cell proliferation was evaluated in lead acetate- and/or si-CCL20 transfected fibroblasts. (e-h) After si-CCL20 transfection, cells were exposed to lead acetate. Then, the effects on hydroxyproline contents (e), fibrosis-associated gene transcripts (f) and protein expression (g, h) were subsequently determined. *P < 0.05 vs. control group. ^#^*P* < 0.05 vs. LA-treated group.

As shown in Figure 2a, transfection with three si-CCL20 dramatically restrained the mRNA transcript of CCL20, concomitant with a decrease in CCL20 protein expression (Figure 2b). Moreover, CCL20 knockdown inhibited lead acetate (LA)-induced CCL20 release (Figure 2c). Intriguingly, lead acetate exposure increased cell proliferation, which was reversed after CCL20 down-regulation (Figure 2d). Furthermore, blockage of CCL20 reduced lead acetate-increased hydroxyproline contents in supernatant from renal fibroblasts (Figure 2e). Further analysis confirmed the elevation of fibrosis-related protein transcript (figure 2f) and expression (Figure 2g and 2h) in lead acetate-stimulated fibroblasts, including CCR6, TGF-β1, FN and collagen I. Notably, CCL20 knockdown inhibited CCL20 expression and reversed lead acetate-induced above increases (figure 2f-2h).

### Identification of miR-143-5p that directly targets CCL20

**Figure 3.**
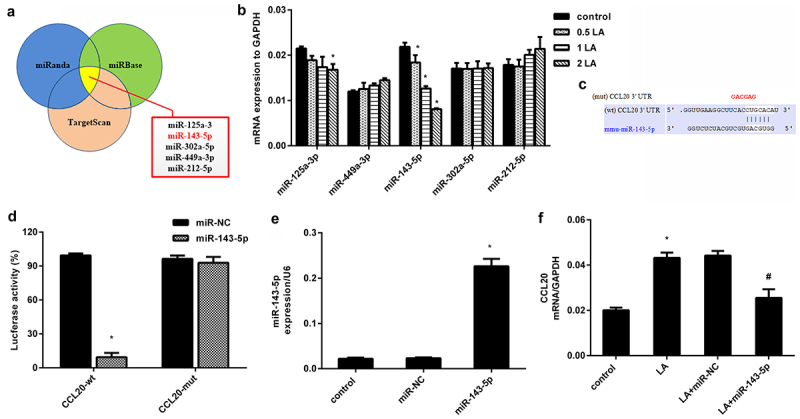
Identification of miR-143-5p that directly targets CCL20. (a) Prediction of miRNAs targeting CCL20 by three bioinformatics tools (TargetScan, miRWalk and miRanda). The five top potential miRNAs were shown in red box. (b) qRT-PCR was used to determine the expression of five miRNAs in fibroblasts under the indicated doses of lead acetates (LA). (c) The predicted binding site of miR-143-5p in the 3’-UTR of CCL20. (d) Dual luciferase reporter assay system was used to evaluate the targeted correlation between CCL20 and miR-143-5p. (e) Cells were transfected with miR-143-5p mimics or miR-NC. Then, the expression of miR-143-5p was analyzed by qRT-PCR. (f) The mRNA levels of CCL20 were determined in fibroblasts treated with lead acetate and miR-143-5p transfections. *P < 0.05 vs. control group. ^#^*P* < 0.05 vs. LA-treated group.

As shown in Figure 3a, we predicted the possible miRNAs that target CCL20 using bioinformatics tools (TargetScan, miRWalk and miRanda) and selected five top potential miRNAs, including miR-125a-3, miR-143-5p, miR-302a-5p, miR-449a-3p, and miR-212-5p. To better select these miRNAs, we determined their expression in lead acetate-treated cells and verified dose-dependent decrease in miR-143-5p expression in renal fibroblasts exposed to lead acetate (Figure 3b). The expression of miR-125a-3 was decreased only when cells were treated with 2 μM lead acetate, and no notable changes were validated in other miRNAs (Figure 3b). As predicted by TargetScan, a potential binding site for miR-143-5p was identified in the 3' UTR of CCL20 (Figure 3c). Further dual-luciferase system substantiated that transfection with miR-143-5p mimic suppressed luciferase activity in the presence of CCL20-3' UTR (wt) co-transfection, but did not have a similar effect on miR-143-5p and CCL20-3' UTR (mut)-treated groups (Figure 3d). Moreover, miR-143-5p mimics transfection induced approximately 9.6-fold increase in miR-143-5p expression relative to the control groups (Figure 3e). Interestingly, miR-143-5p overexpression muted lead acetate-induced CCL20 transcripts (figure 3f). Therefore, CCL20 may serve as a direct target of miR-143-5p.

### miR-143-5p antagonizes lead acetate-induced dysfunction of renal interstitial fibroblasts by targeting CCL20

**Figure 4.**
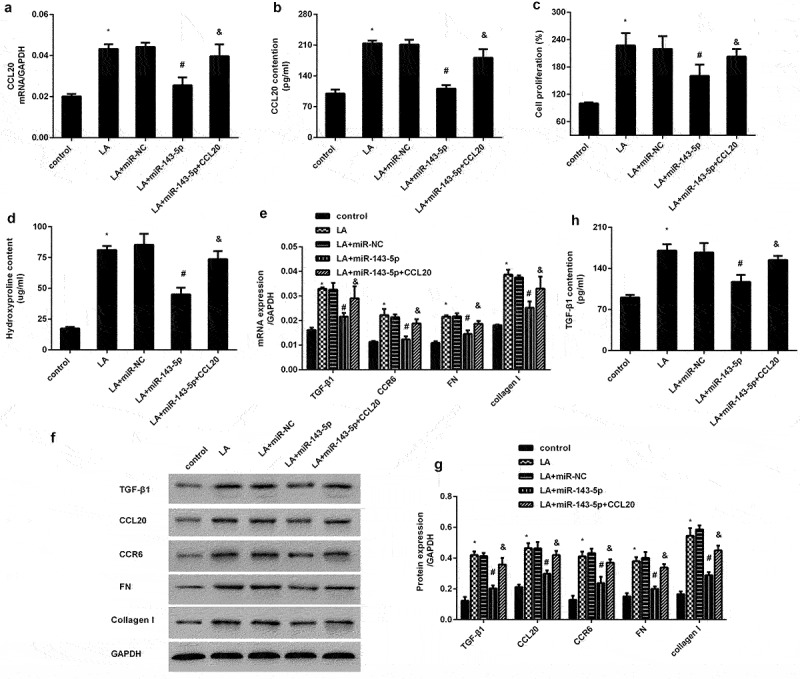
Elevation of miR-143-5p reverses lead acetate-induced proliferation and dysfunction of renal fibroblasts by targeting CCL20. Fibroblasts were transfected with miR-143-5p mimics or recombinant CCL20 plasmids, prior to lead acetate exposure. Then, the transcripts (a) and production of CCL20 (b) were analyzed by qRT-PCR and ELISA assay. The subsequent effects on cell proliferation (c), hydroxyproline levels (d), ECM-related gene expression (e-g) and TGF-β1 (h) were detected. *P < 0.05 vs. control group. ^#^*P* < 0.05 vs. LA-treated group. ^&^*P* < 0.05 vs. LA+miR-143-5p-treated group.

To further elucidate the mechanism underlying CCL20-mediated dysfunction of renal interstitial fibroblasts triggered by lead exposure, we investigated the function of miR-143-5p during these processed. Of note, miR-143-5p overexpression significantly inhibited lead acetate-induced increase in CCL20 transcripts (Figure 4a) and releases (Figure 4b). Nevertheless, CCL20 elevation overturned miR-143-5p-mediated inhibition in CCL20 transcripts and production (Figure 4a and 4b). Additionally, up-regulation of miR-143-5p antagonized lead acetate-evoked increases in cell proliferation (Figure 4c) and hydroxyproline levels (Figure 4d), which were counteracted when cells were transfected with CCL20 recombinant vectors (Figure 4c and 4d). Further analysis revealed the suppressive role of miR-143-4p enhancement in lead acetate-induced up-regulation of CCR6, TGF-β1, FN and collagen I (Figure 4e-4g). Concomitantly, the inhibitory effects of miR-143-5p on TGF-β1 production were also verified in lead acetate-treated fibroblasts (Figure 4h). However, overexpression of CCL20 offset the suppressive effects of miR-143-5p on lead acetate-triggered pro-fibrosis protein expression.

### miR-143 targets CCL20 to affect Smad2/3 and AKT signaling in lead acetate-treated renal interstitial fibroblasts

**Figure 5.**
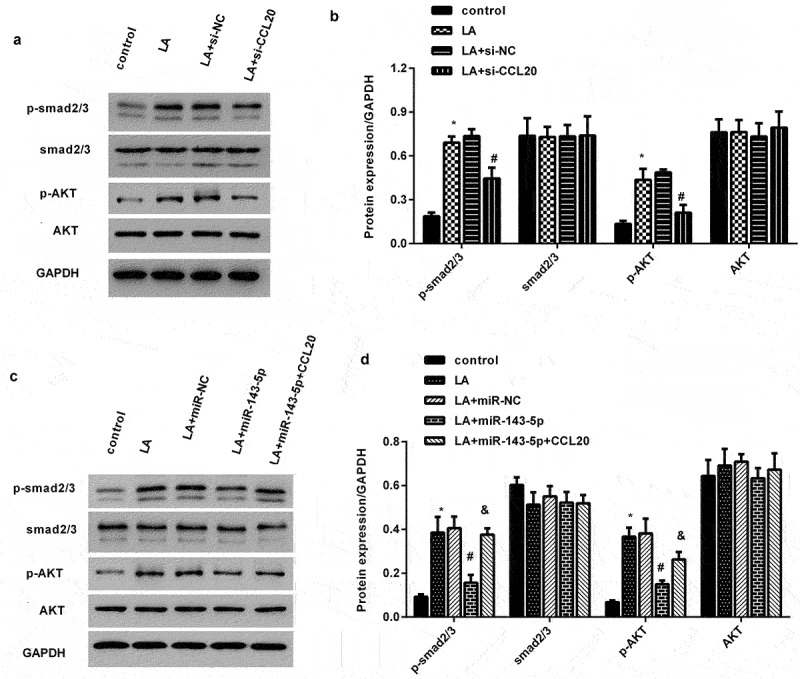
miR-143-5p regulates lead acetate-activated the pathways of the Smad2/3 and AKT by targeting CCL20. (a, b) Cells were treated with si-CCL20 transfection and lead acetate exposure. Then, the expression of p-Smad2/3, Smad2/3, p-AKT and AKT were determined by western blotting. The corresponding quantify of protein bands was performed by Image J software. (c, d) The activation of the Smad2/3 and AKT signaling was analyzed in lead acetate-stimulated cells that were pre-transfected with miR-143-5p mimics or CCL20 plasmids. *P < 0.05 vs. control group. ^#^*P* < 0.05 vs. LA-treated group. ^&^*P* < 0.05 vs. LA+miR-143-5p-treated group.

Accumulating evidence indicates the critical function of Smad2/3 and AKT signaling in renal fibrosis progression [22,23]. As shown in Figure 5a and 5b, exposure to lead acetate enhanced the expression of p-Smad2/3 and p-AKT; however, these increased expression was inhibited after CCL20 knockdown. Importantly, elevation of miR-143-5p exhibited a similar inhibition effect on p-Smad2/3 and p-AKT expression (Figure 5c and 5d). However, CCL20 plasmids transfection muted the inhibitory efficacy of miR-143-5p in lead acetate-mediated activation of Smad2/3 and AKT signaling (Figure 5c and 5d).

### Injection with miR-143 mimics attenuates renal fibrosis progression and CCL20-mediated signaling in lead acetate-induced mice model

**Figure 6.**
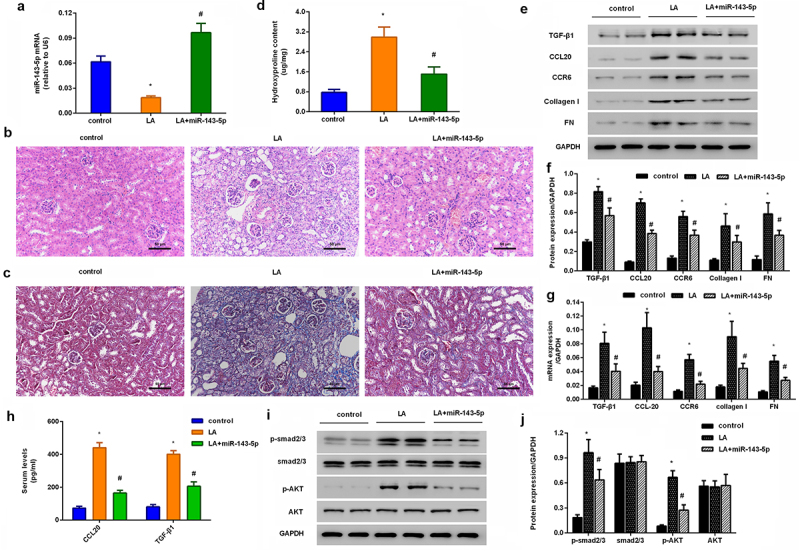
Injection with miR-143-5p alleviates the development of renal fibrosis in lead acetate-treated mice. (a) Mice under lead acetate injected were underwent miR-143-5p overexpression. Then, the expression of miR-143-5p was analyzed. (b, c) Kidney fibrosis was evaluated by H&E (b) and Masson detection (c) Scale bar = 50 μm. (d) The contents of hydroxyproline were determined in plasma from mice. (e, f) Western assay was then performed to detect the protein expression of CCL20, TGF-β1, CCR6, Collagen I and FN. (g) qRT-PCR was performed to determine the mRNA levels of fibrosis-related gene expression. (h) ELISA assay was used to measure plasma levels of CCL20 and TGF-β1. (i, j) Effects on the activation of the Smad2/3 and AKT pathways were analyzed. *P < 0.05 vs. control group. ^#^*P* < 0.05 vs. LA-treated group.

*In vivo*, a considerable downregulation of miR-143-5p was observed in renal tissues obtained from mice injected with lead acetate; nevertheless, miR-143-5p injection noticeably enhanced its expression in mice (Figure 6a). Intriguingly, H&E staining revealed renal structural injury in lead acetate-treated mice (Figure 6b). Moreover, abundant collagen fiber streaks were observed in lead acetate-injected mice by Masson’s staining (Figure 6c). Noticeably, miR-143-5p elevation alleviated lead acetate-induced renal damage and fibrosis degree (Figure 6b and 6c). Furthermore, in contrast to the control groups, lead acetate injection increased hydroxyproline contents in plasma (Figure 6d) and expression of fibrosis-related proteins (CCR6, TGF-β1, FN, and collagen I) (Figure 6e-6g) in renal tissues, which were reversed after miR-143-5p overexpression. Additionally, enhancement of miR-143-5p also decreased lead acetate-induced CCL20 and TGF-β1 expression and levels in plasma (Figure 6e-6g), concomitant with the inhibition in the activation of Smad2/3 and AKT pathways (Figure 6h and 6i).

## Discussion

Chronic kidney disease (CKD) is a major public health concern affecting approximately half of adults above the age of 70 years and 13% of the global population [1,5]. CKD develops into renal fibrosis that is a terminal manifestation and irreversible pathway for CKD progression to renal failure. Lead is a poisonous heavy metal that usually causes pathophysiological toxicity and fibrotic progression in many living organs including kidney [7,8]. However, the molecular mechanisms underlying lead-related nephrotoxicity remain unclear. Herein, we isolated primary mouse renal interstitial fibroblasts and firstly performed gene expression profiles in lead acetate-treated renal interstitial fibroblasts using RNA-seq, and identified CCL20 as one of the most predominantly up-regulated genes. Similarly, qRT-PCR and western blotting corroborated the obvious up-regulation of CCL20 in lead acetate-treated fibroblasts. Therefore, these findings indicate a potential role of CCL20 in fibrotic kidney disease.

Epidemiological and toxicological studies have suggested that environmental lead contamination is a critical cause of nephrotoxicity [7,11]. Tubulointerstitial fibrosis is a common pathological basis for the development of CKD to end-stage renal diseases. During the progression, lead exposure activates fibroblasts to transform from the resting state into the proliferative state to synthesize excessive ECM, such as collagen I and fibronectin (FN), resulting in renal fibrosis [4,24]. Therefore, excessive interstitial fibroblast proliferation and ECM deposition are recognized as the key pathogenic contributors to renal interstitial fibrosis. Noticeably, increased serum lead level has been proven to be independently associated with an increased risk of advanced fibrosis [9]. In this study, analogously with our previous findings [17], lead exposure induced fibroblast proliferation and abundant ECM-related collagen I, fibronectin expression. Furthermore, lead acetate increased hydroxyproline production, which exists almost exclusively in collagen and can indirectly reveal the content of collagen. Stimulation with lead acetate increased CCR6 and TGF-β expression, which are the proverbial mediators to trigger fibrosis [25–27]. Intriguingly, CCL20 knockdown reversed lead acetate-induced proliferation, hydroxyproline levels, and pro-fibrotic protein expression. Chemokine CCL20 is a pleiotropic driver of a variety of pathogenic processes, such as injury and inflammation. Emerging evidence confirms the key roles of CCL20 in various fibrotic processes, including cardiac and liver fibrosis [14,15,28]. Thus, these data suggest a key function of CCL20 in lead-induced pro-fibrotic process in renal interstitial fibroblasts.

MicroRNAs (miRs) rank as the critical regulators of gene expression by controlling mRNAs expression in various organs. Multiple studies have shown that miRNAs exert key roles in many biological processes, including renal injury and fibrosis [12,29]. Next, we selected five top potential miRNAs targeting CCL20 by bioinformatics tools, and found that only miR-143-5p expression decreased in a dose-dependent manner in lead acetate-treated fibroblasts. Additionally, luciferase reporter system had identified CCL20 as a direct target of miR-143-5p that dramatically suppressed CCL20 transcripts. These data indicate that miR-143-5p may serve as a direct target of CCL20. More importantly, miR-143-5p overexpression antagonized lead acetate-induced fibroblast proliferation and ECM deposition by decreasing ECM-related protein expression, which was reversed by CCL20 elevation, suggesting that miR-143-5p may target CCL20 to inhibit lead-evoked fibroblast proliferation and pro-fibrotic efficacy. Intriguingly, loss of miR-143/145 cluster leads to hydronephrosis [13]. Emerging evidence confirms that miR-143 suppresses mesangial cell proliferation and fibrosis, suggesting the key role of miR-143 in diabetic kidney disease progression [30,31]. Noticeably, miR-143 ameliorates the development of hepatic fibrosis [32]. More importantly, the present study demonstrated that miR-143-5p injection ameliorated the progression of renal fibrosis in lead acetate-treated mice.

We next substantiated that CCL20 suppression blocked the activation of smad2/3 and AKT signaling evoked by lead acetate exposure. TGF-β is a predominant contributor influencing fibrosis in kidneys and other organs, and its pro-fibrotic efficacy is mainly regulated by the activation of the Smad2/3 signaling [22,33]. Additionally, abundant evidence has implicated the conventional TGF-β/Smad and non-canonical TGF-β/PI3K/AKT pathways in various renal diseases, including renal fibrosis [34–37]. Of interest, elevation of miR-143-5p inhibited lead acetate-induced activation of the Smad2/3 and AKT pathways *in vitro*, which was reversed by CCL20 overexpression. Therefore, targeting CCL20 by miR-143 may offset lead-induced pro-fibrotic Smad2/3 and AKT pathway activation. Noticeably, miR-143-5p injection also inhibited the activation of Smad2/3 and AKT pathway and the subsequent expression of ECM-related proteins *in vivo* in lead acetate-treated mice. However, how CCL20 regulates smad2/3 or AKT during this process? Whether CCL20 regulates the Smad2/3 and AKT pathways by TGF-β. Are other signaling involved in CCL20-mediated anti-fibrotic process? All of these issues will be further investigated in the next plan.

## Conclusions

The current study revealed that CCL20 was predominantly expressed in lead acetate-treated renal interstitial fibroblasts. Moreover, targeting CCL20 by miR-143-5p attenuated lead acetate-induced fibroblast proliferation, hydroxyproline production and ECM deposition that involved in the activation of the Smad2/3 and AKT pathways. Notably, miR-143-5p administration ameliorated lead acetate-induced renal fibrosis *in vivo*. Therefore, these findings may first highlight an interesting fact that targeting CCL20 by miR-143-5p may alleviate renal fibrosis by regulating fibroblast proliferation and pro-fibrotic efficacy. Therefore, the current data could be used to develop a potential therapeutic strategy for fibrotic kidney disease caused by environmental lead contamination.

## Supplementary Material

Supplemental Material
