# Supplementary material for: Targeting CC chemokine ligand (CCL) 20 by miR-143-5p alleviate lead poisoning-induced renal fibrosis by regulating interstitial fibroblasts excessive proliferation and dysfunction
Source: Bioengineered. 2022 Apr 29;13(4):11156–68. doi: 10.1080/21655979.2022.2062106 (PMC9208521; doi:10.1080/21655979.2022.2062106)
Supplement: Supplemental Material [file KBIE_A_2062106_SM2482.zip › supplementary/Certificate_of_editing.pdf]

CERTIFICATE OF ENGLISH EDITING

This document certifies that the paper listed below has been edited to ensure that the language is clear and free of errors. The edit was performed by professional editors at Editage, a division of Cactus Communications, in cooperation with Taylor & Francis Group. The intent of the author's message was not altered in any way during the editing process. The quality of the edit has been guaranteed, with the assumption that our suggested changes have been accepted and have not been further altered without the knowledge of our editors.

Title

Targeting CCL20 by miR-143-5p alleviates lead poisoning-induced renal fibrosis

Authors

Chen Yu

Order No.

AFNRA_1

EDITINGSERVICES
Supporting Taylor & Francis authors

Signature

Vikas Narang

Vikas Narang,
Chief Operating Officer,
Editage

editage

Date of Issue
March 09, 2022
