# Supplementary figures and images for: Targeting CC chemokine ligand (CCL) 20 by miR-143-5p alleviate lead poisoning-induced renal fibrosis by regulating interstitial fibroblasts excessive proliferation and dysfunction
Source: Bioengineered. 2022 Apr 29;13(4):11156–68. doi: 10.1080/21655979.2022.2062106 (PMC9208521; doi:10.1080/21655979.2022.2062106)

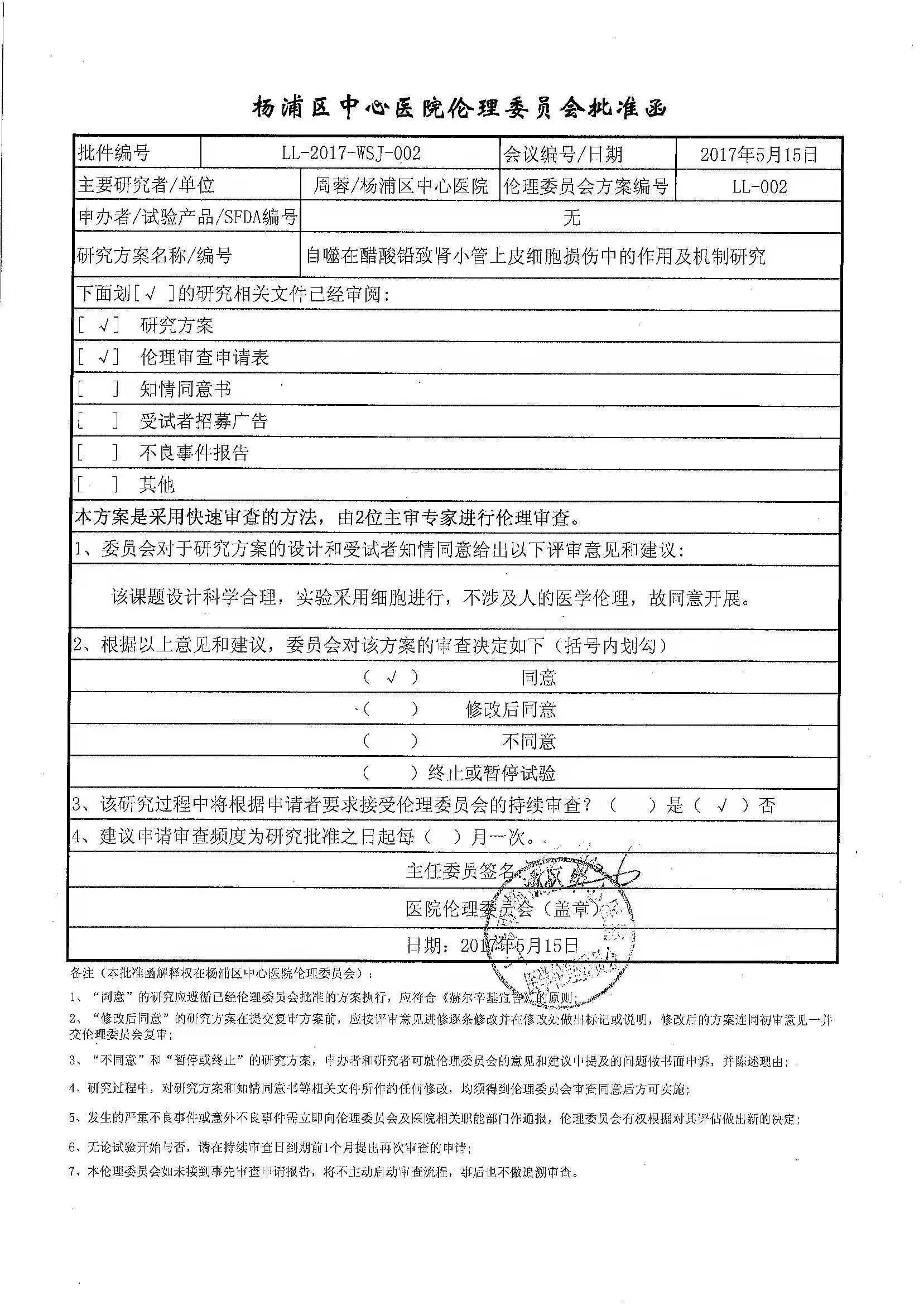

Supplement: Supplemental Material [file KBIE_A_2062106_SM2482.zip › supplementary/ethical approvement.jpg]

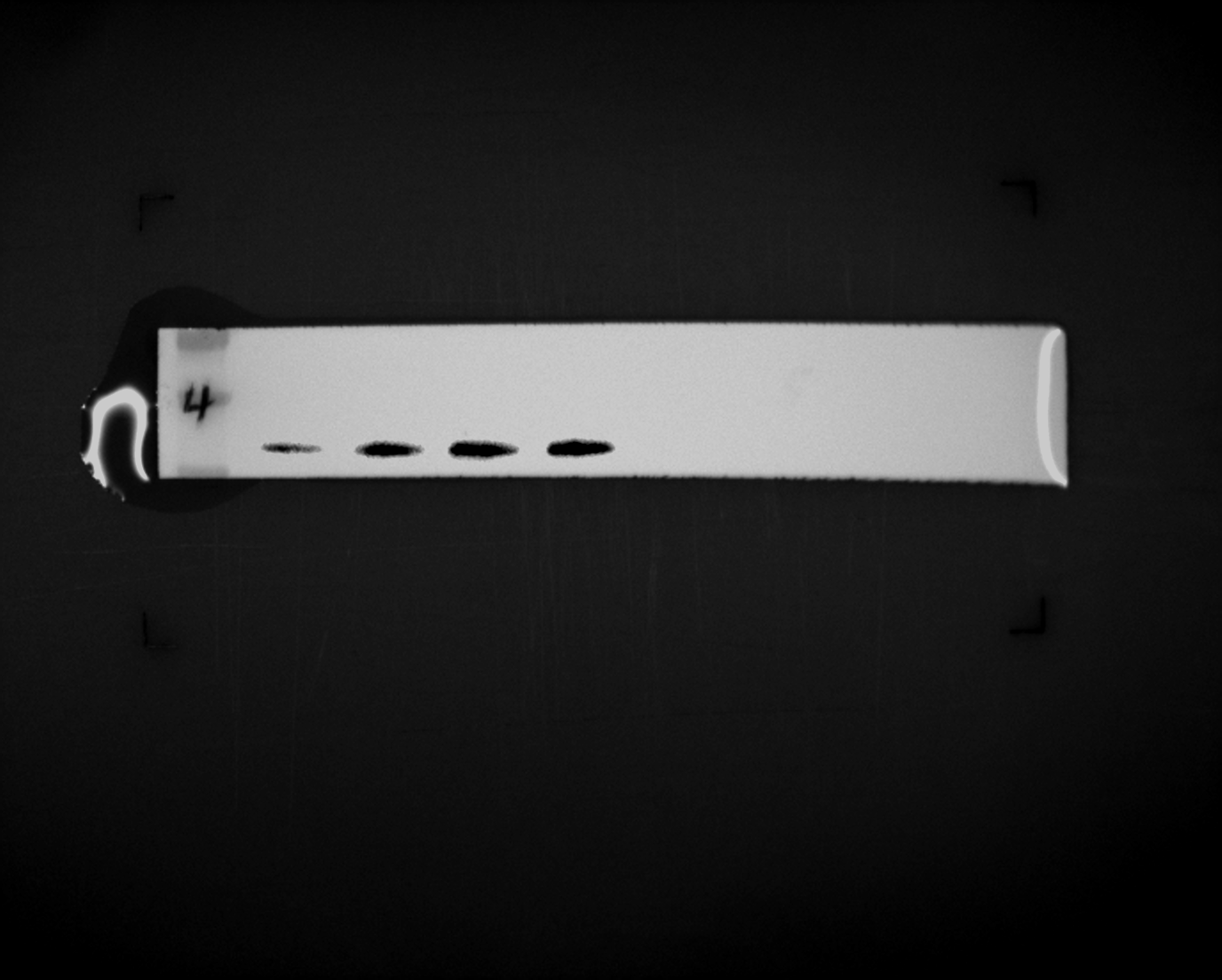

Supplement: Supplemental Material [file KBIE_A_2062106_SM2482.zip › supplementary/Fig1E CCL20.tif]

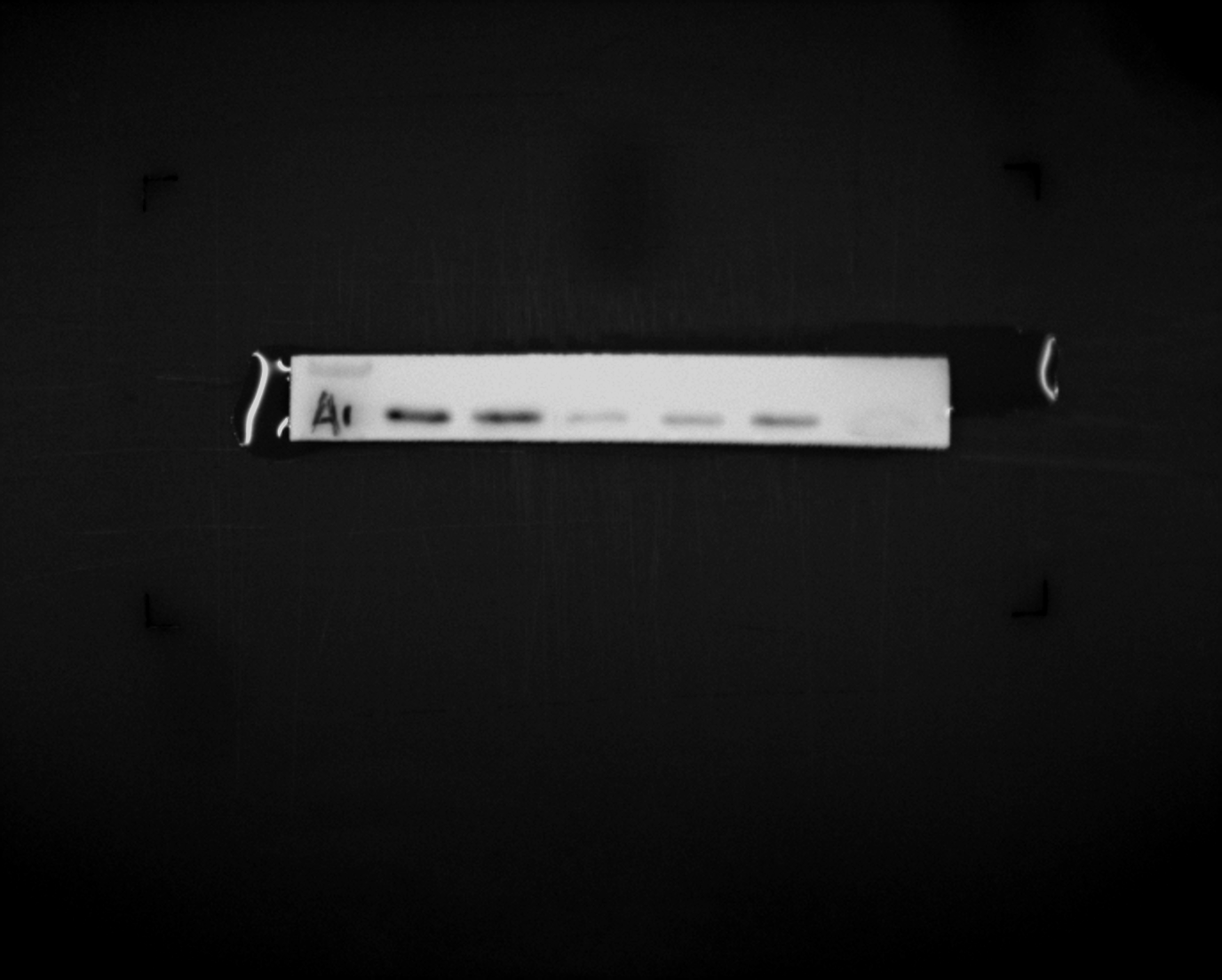

Supplement: Supplemental Material [file KBIE_A_2062106_SM2482.zip › supplementary/Fig2B_CCL20.tif]

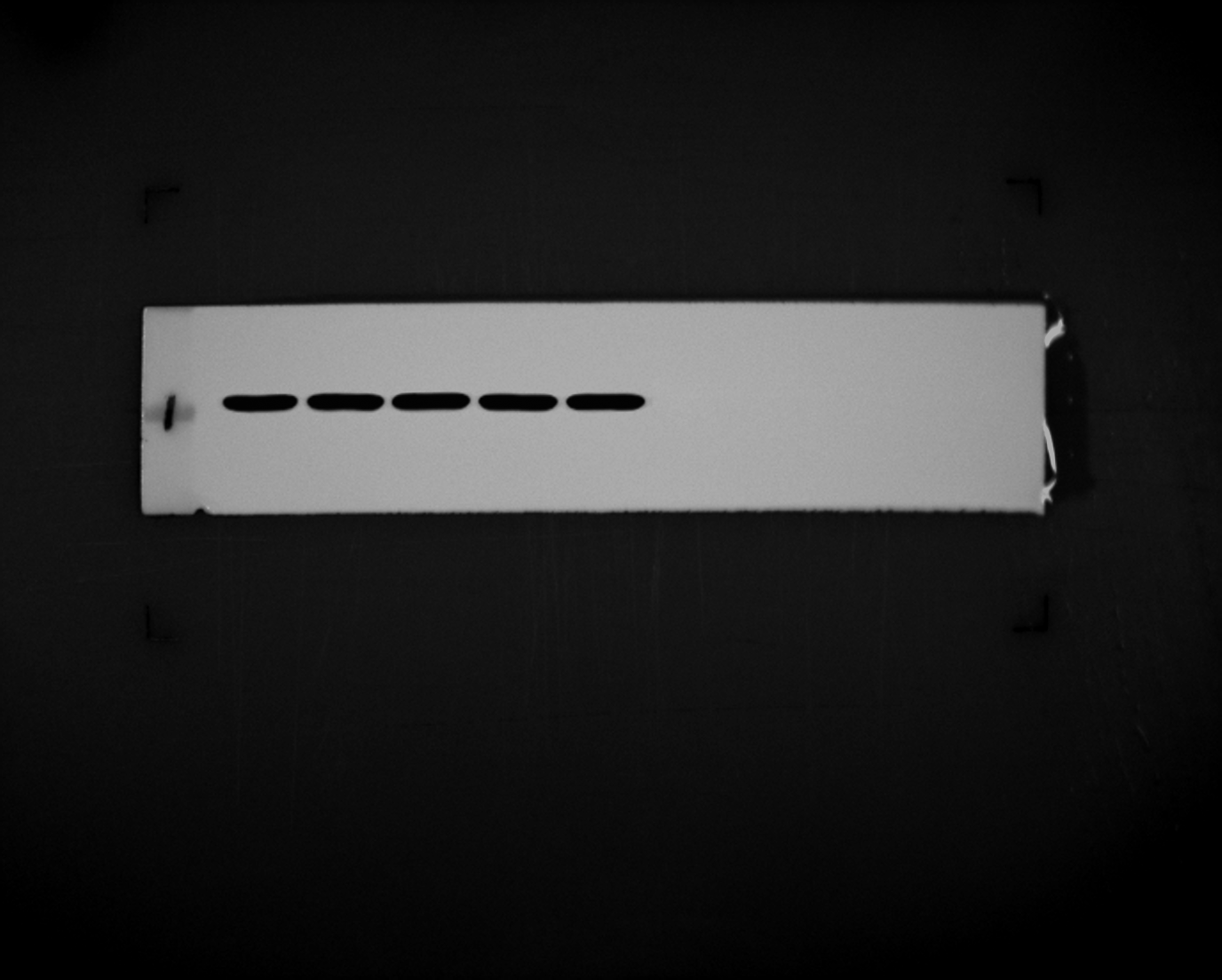

Supplement: Supplemental Material [file KBIE_A_2062106_SM2482.zip › supplementary/Fig2B_GAPDH.tif]

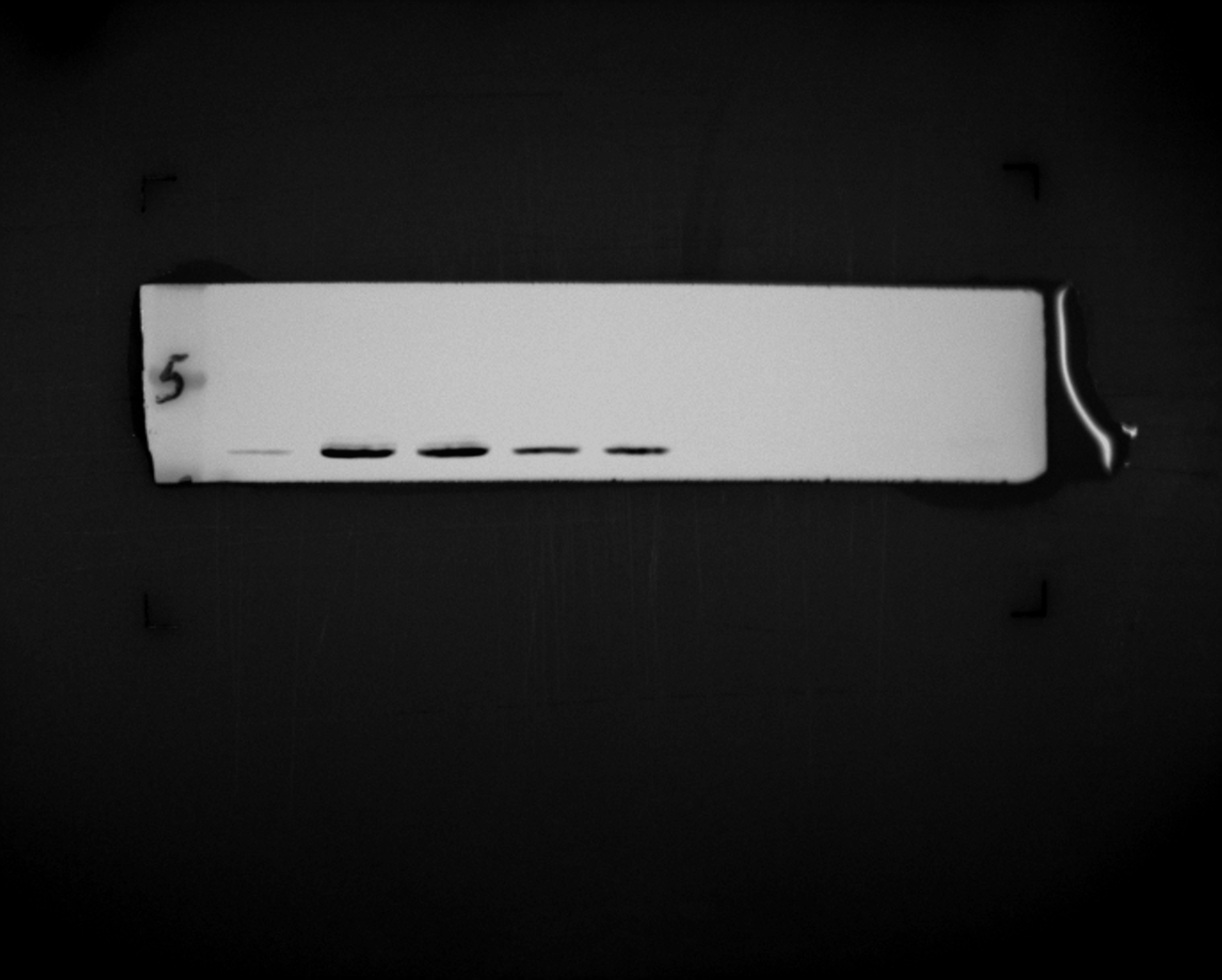

Supplement: Supplemental Material [file KBIE_A_2062106_SM2482.zip › supplementary/Fig2G_CCL20.tif]

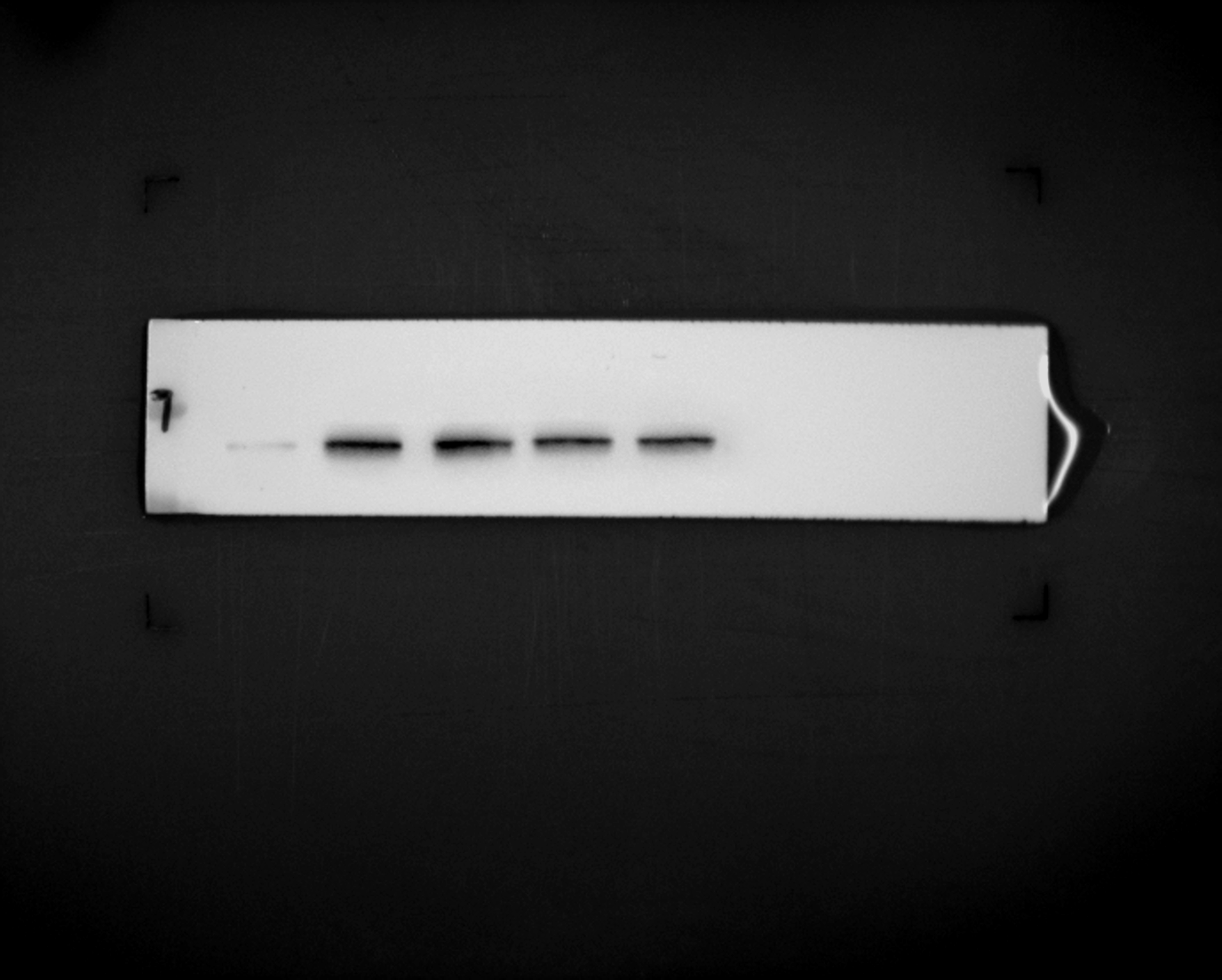

Supplement: Supplemental Material [file KBIE_A_2062106_SM2482.zip › supplementary/Fig2G_CCR6.tif]

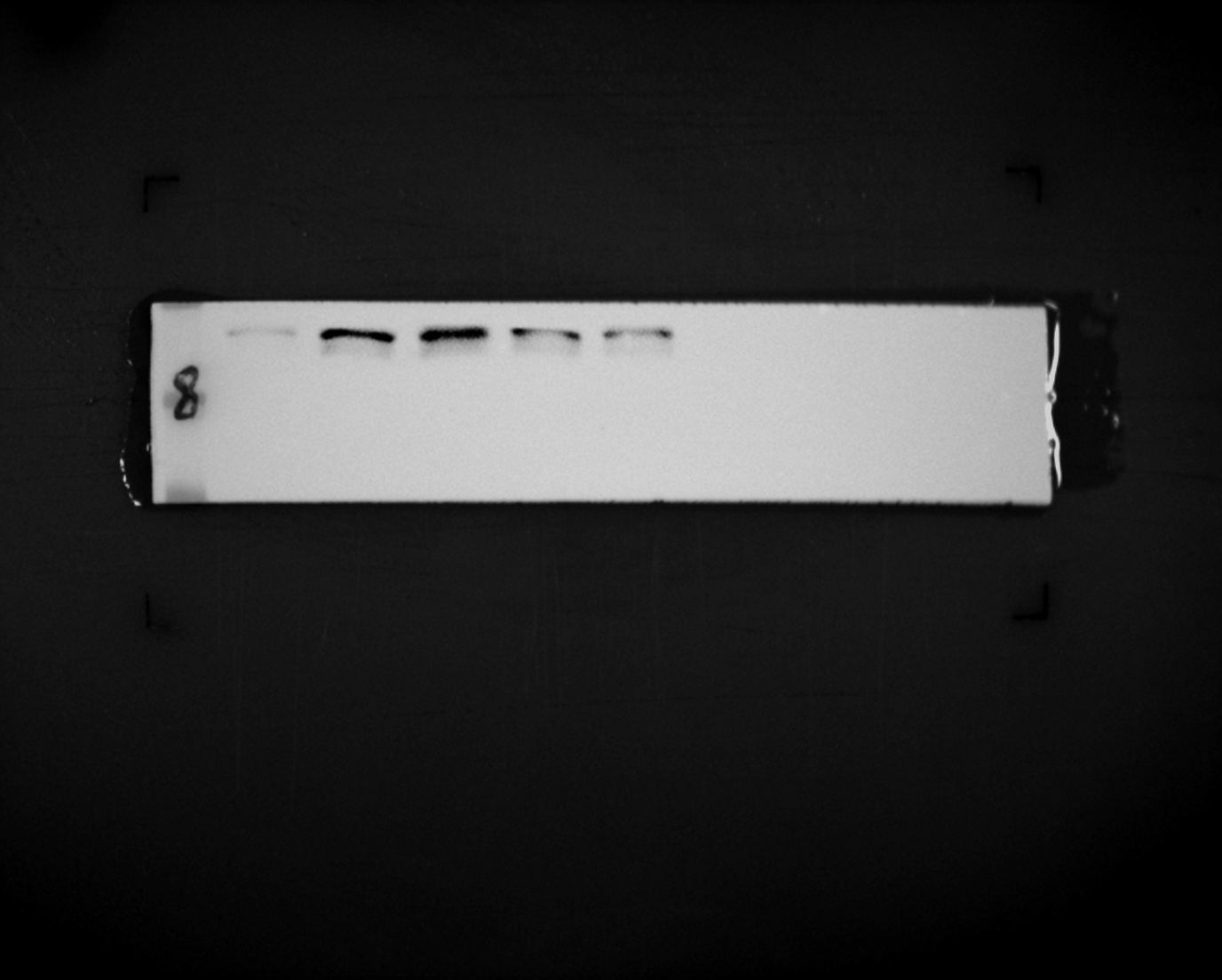

Supplement: Supplemental Material [file KBIE_A_2062106_SM2482.zip › supplementary/Fig2G_CollagenI.tif]

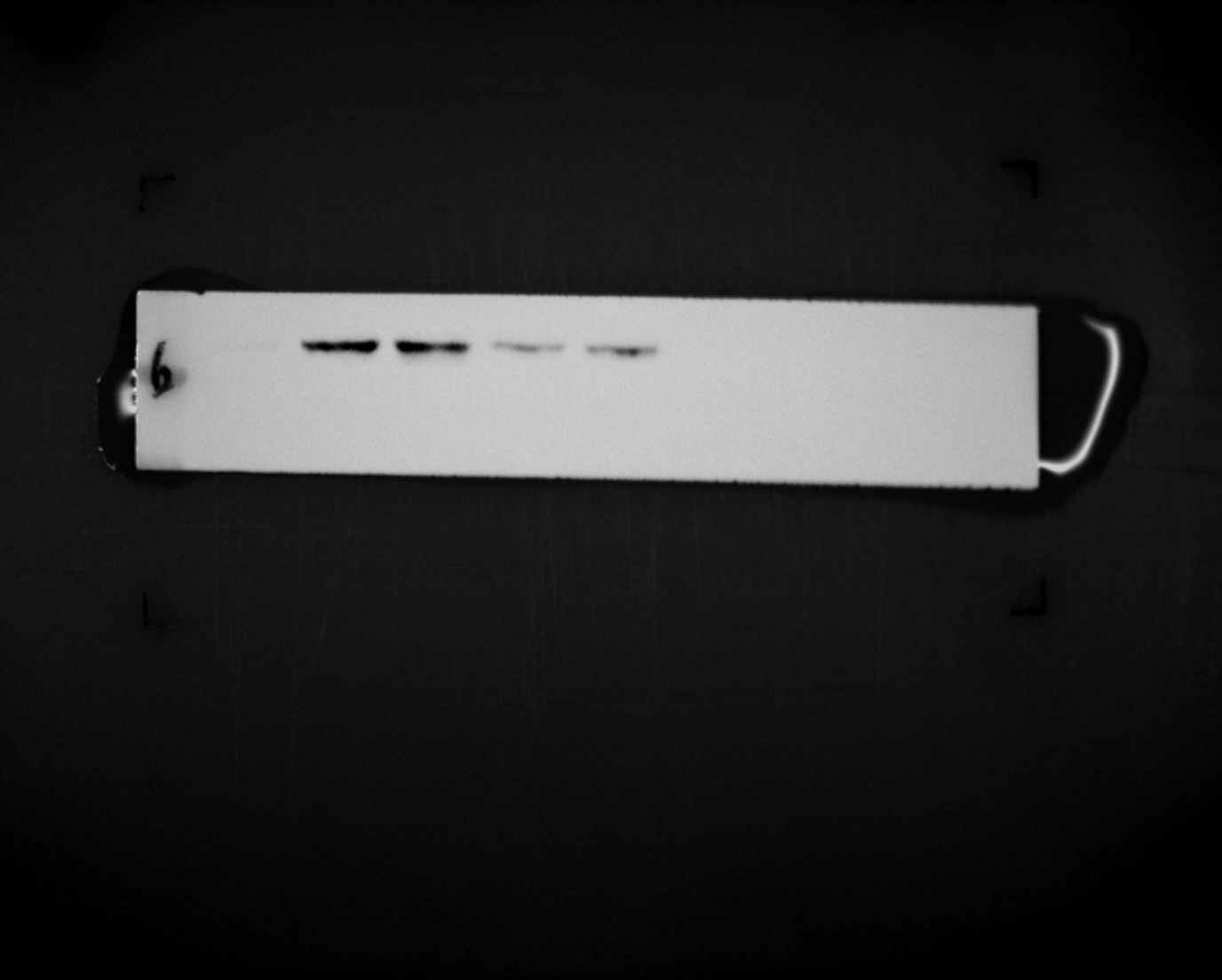

Supplement: Supplemental Material [file KBIE_A_2062106_SM2482.zip › supplementary/Fig2G_FN.tif]

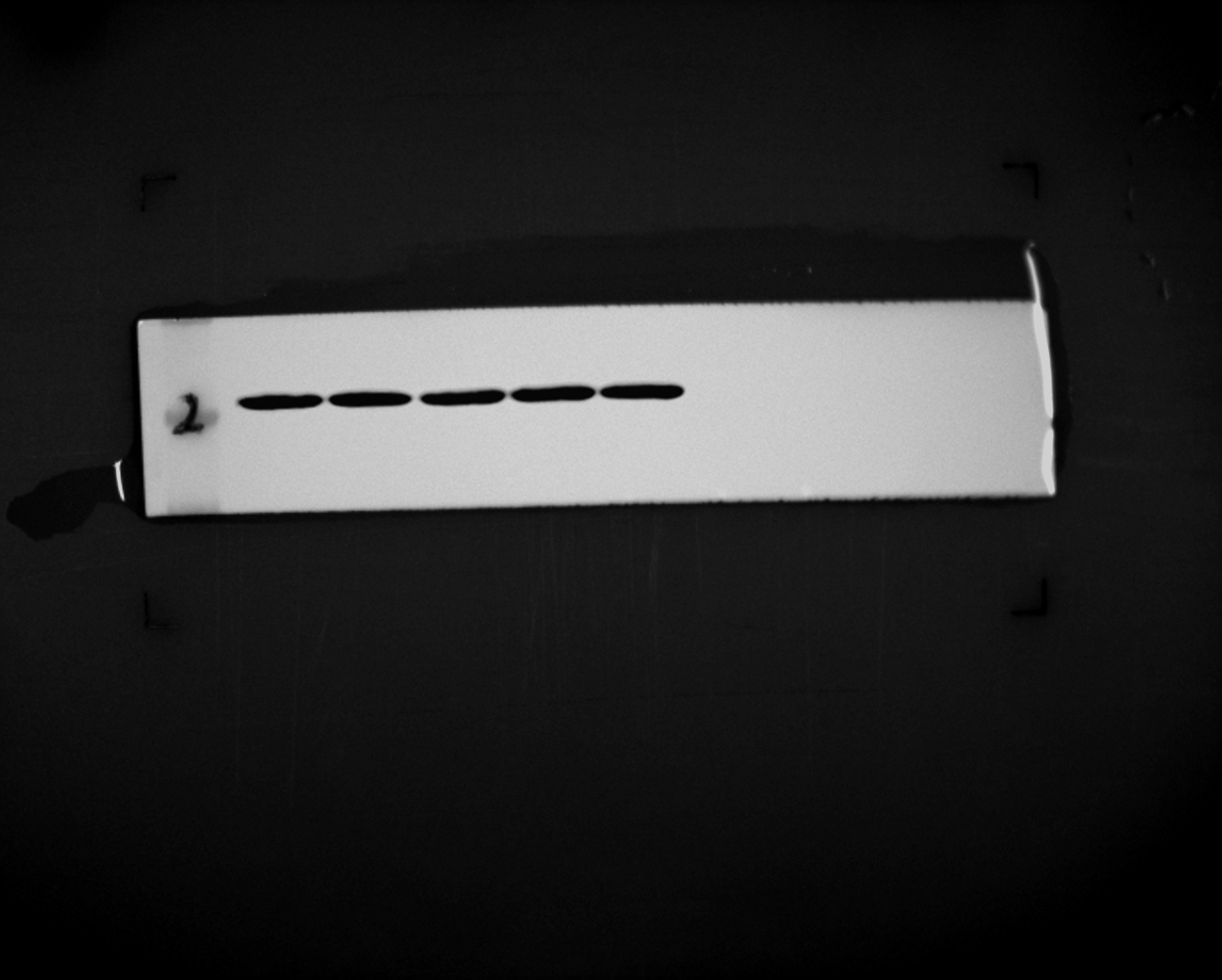

Supplement: Supplemental Material [file KBIE_A_2062106_SM2482.zip › supplementary/Fig2G_GAPDH.tif]

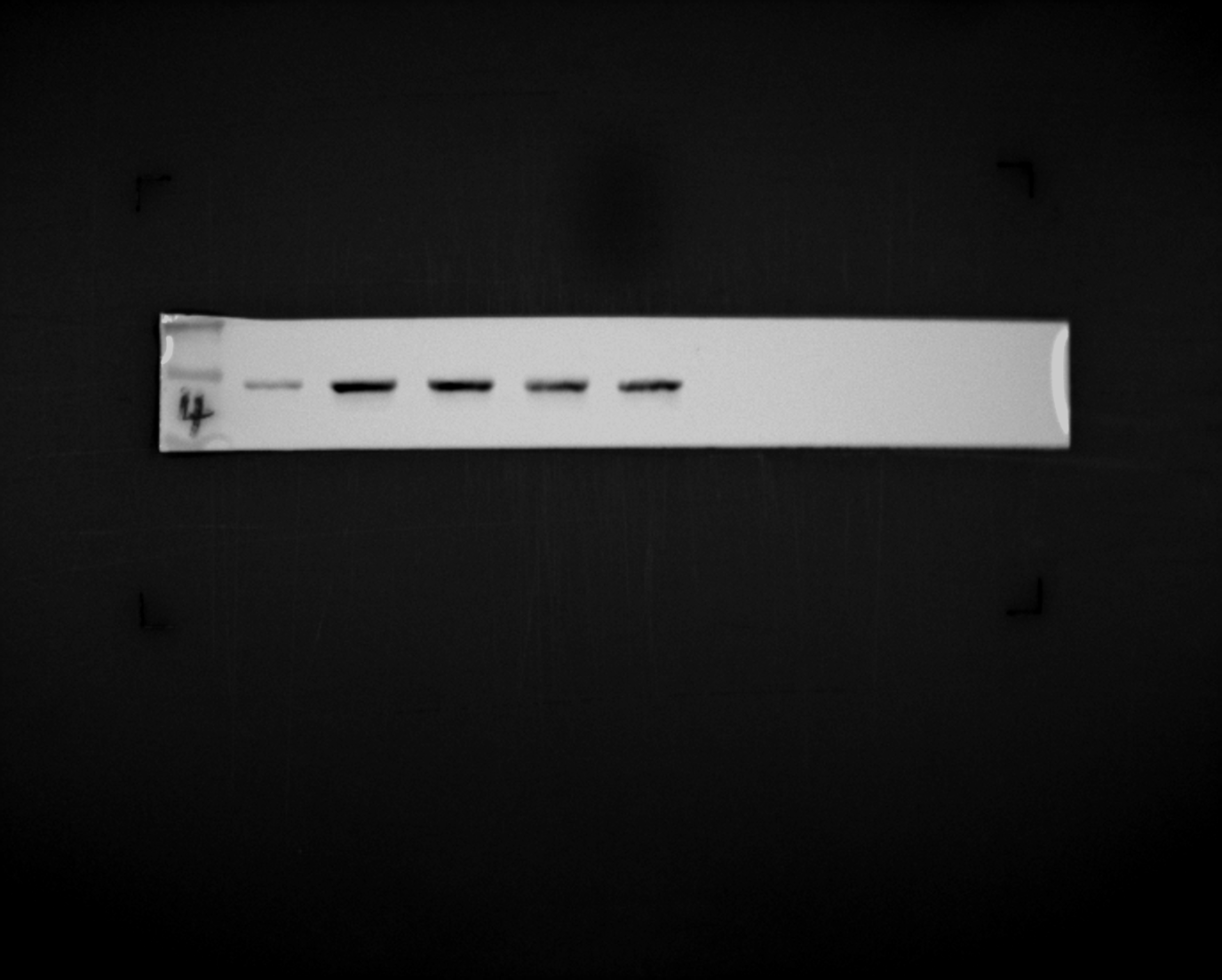

Supplement: Supplemental Material [file KBIE_A_2062106_SM2482.zip › supplementary/Fig2G_TGF_1.tif]

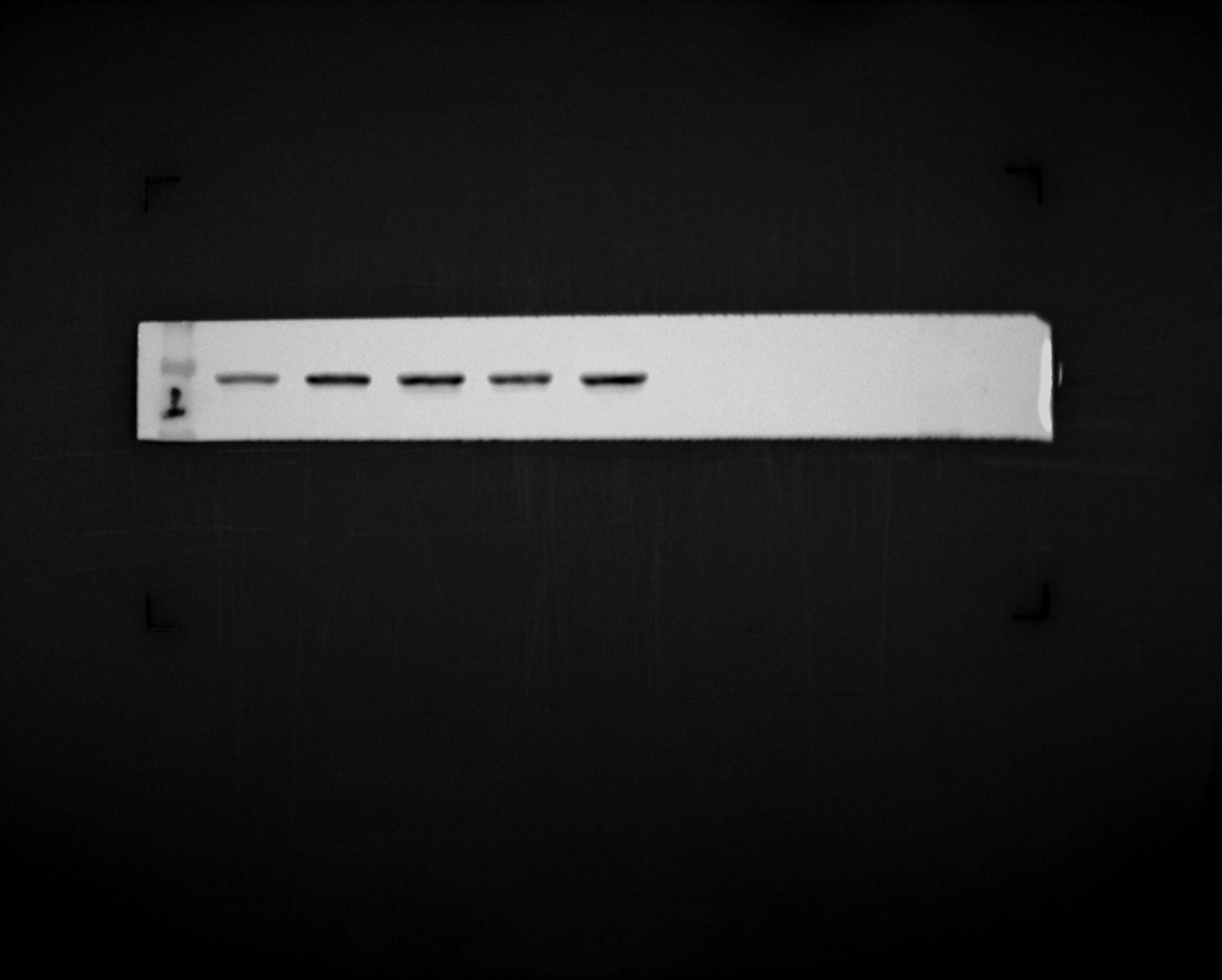

Supplement: Supplemental Material [file KBIE_A_2062106_SM2482.zip › supplementary/Fig4F_CCL20.tif]

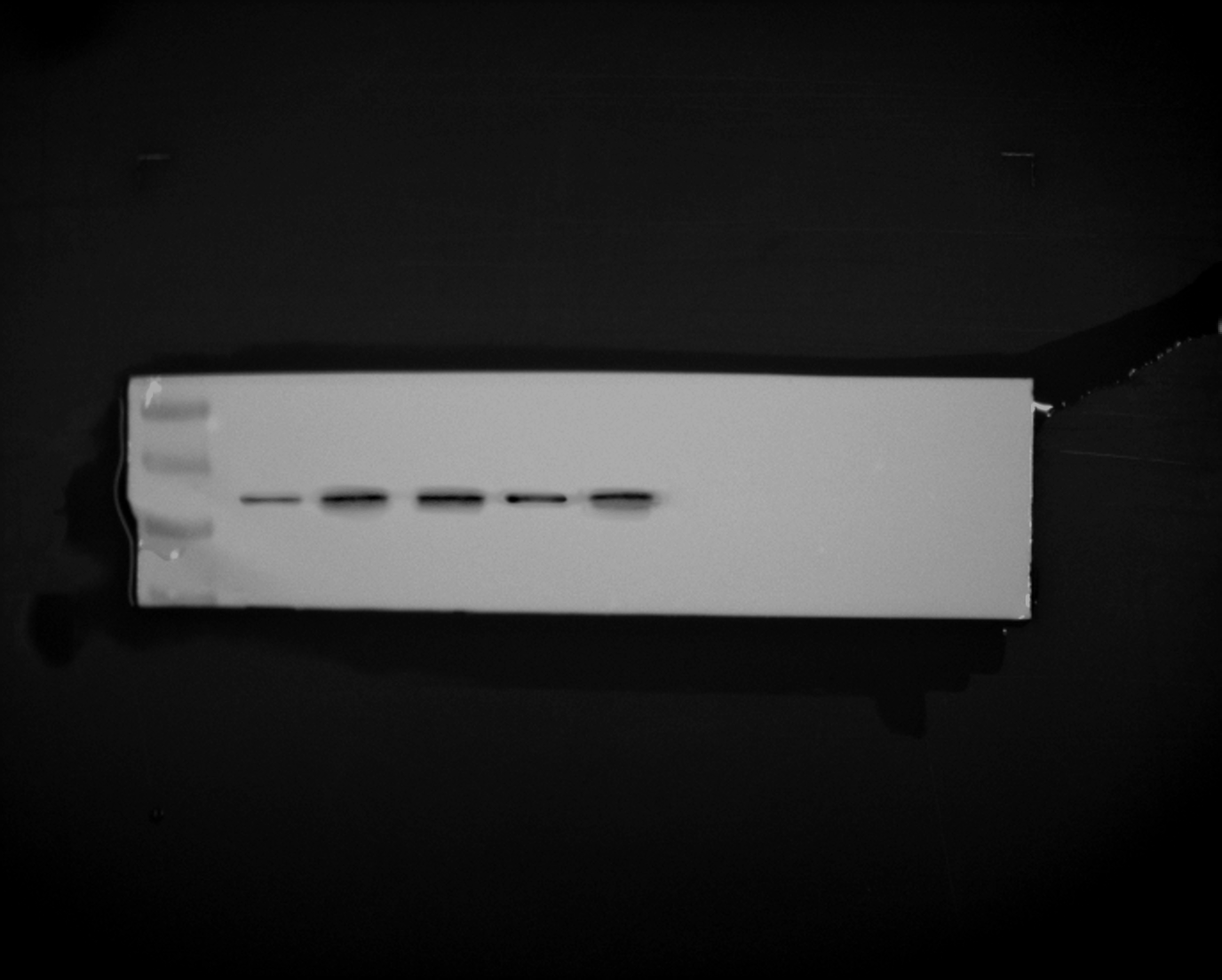

Supplement: Supplemental Material [file KBIE_A_2062106_SM2482.zip › supplementary/Fig4F_CCR6.tif]

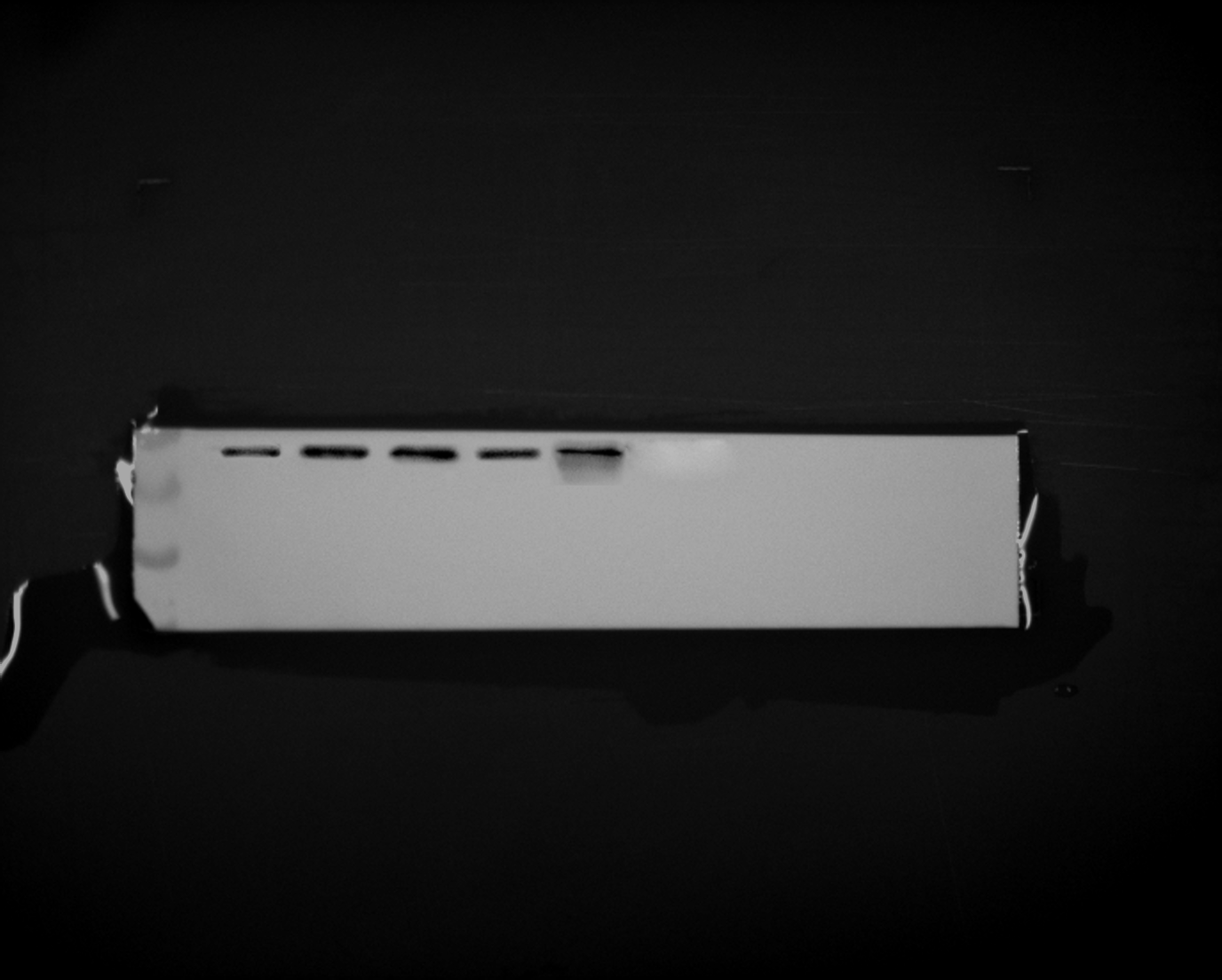

Supplement: Supplemental Material [file KBIE_A_2062106_SM2482.zip › supplementary/Fig4F_Collagen.tif]

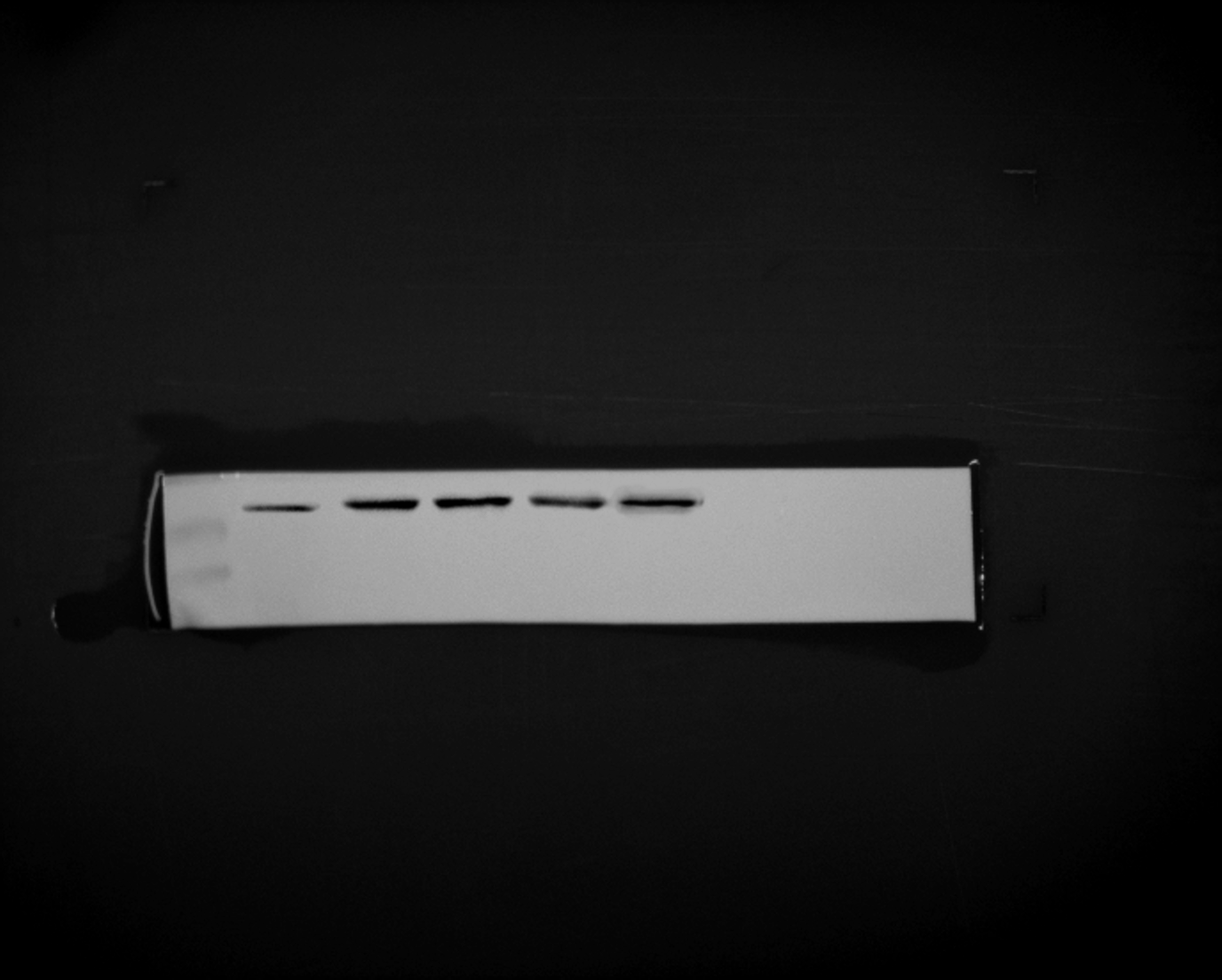

Supplement: Supplemental Material [file KBIE_A_2062106_SM2482.zip › supplementary/Fig4F_FN.tif]

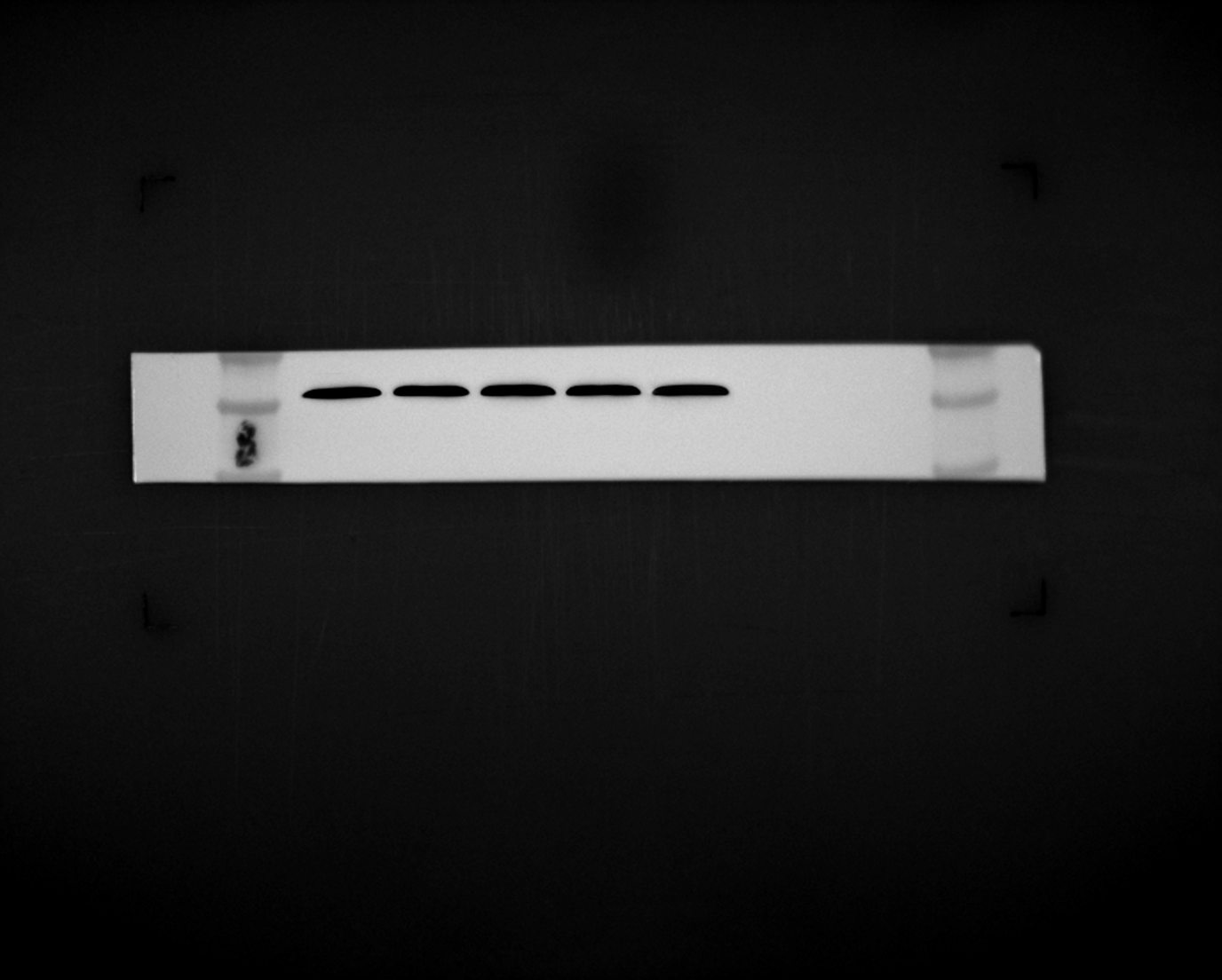

Supplement: Supplemental Material [file KBIE_A_2062106_SM2482.zip › supplementary/Fig4F_GAPDH.tif]

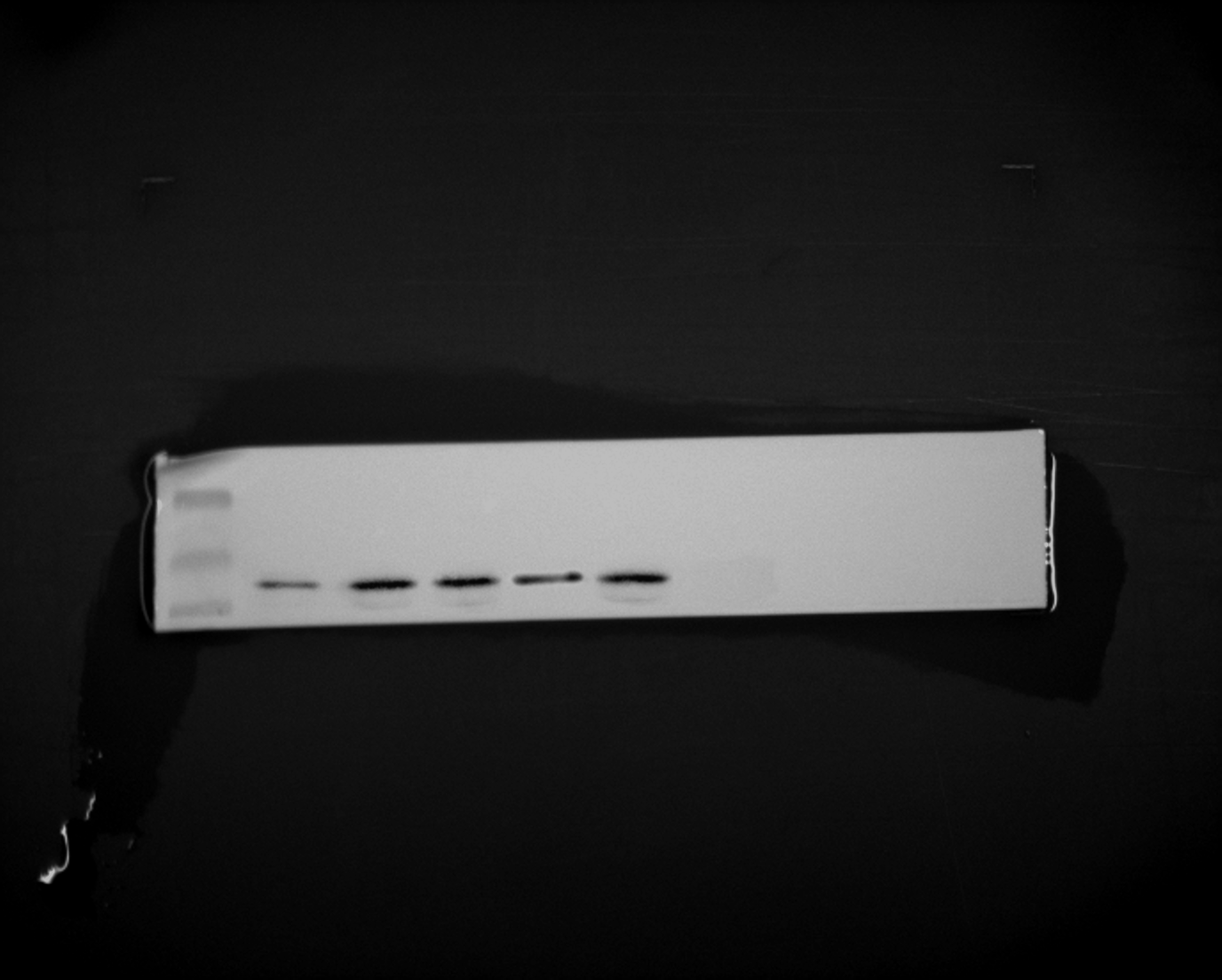

Supplement: Supplemental Material [file KBIE_A_2062106_SM2482.zip › supplementary/Fig4F_TGFB1.tif]

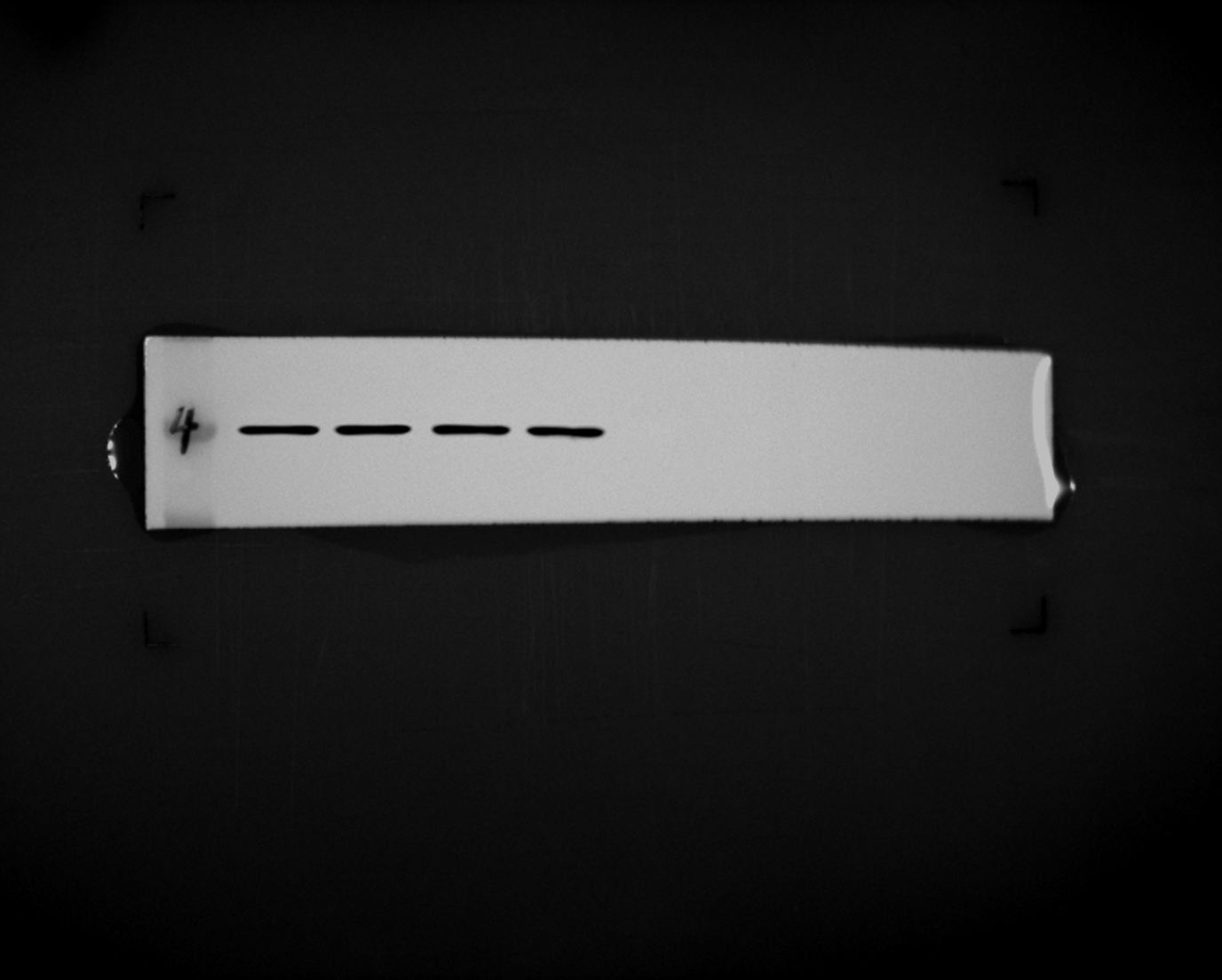

Supplement: Supplemental Material [file KBIE_A_2062106_SM2482.zip › supplementary/Fig5A_AKT.tif]

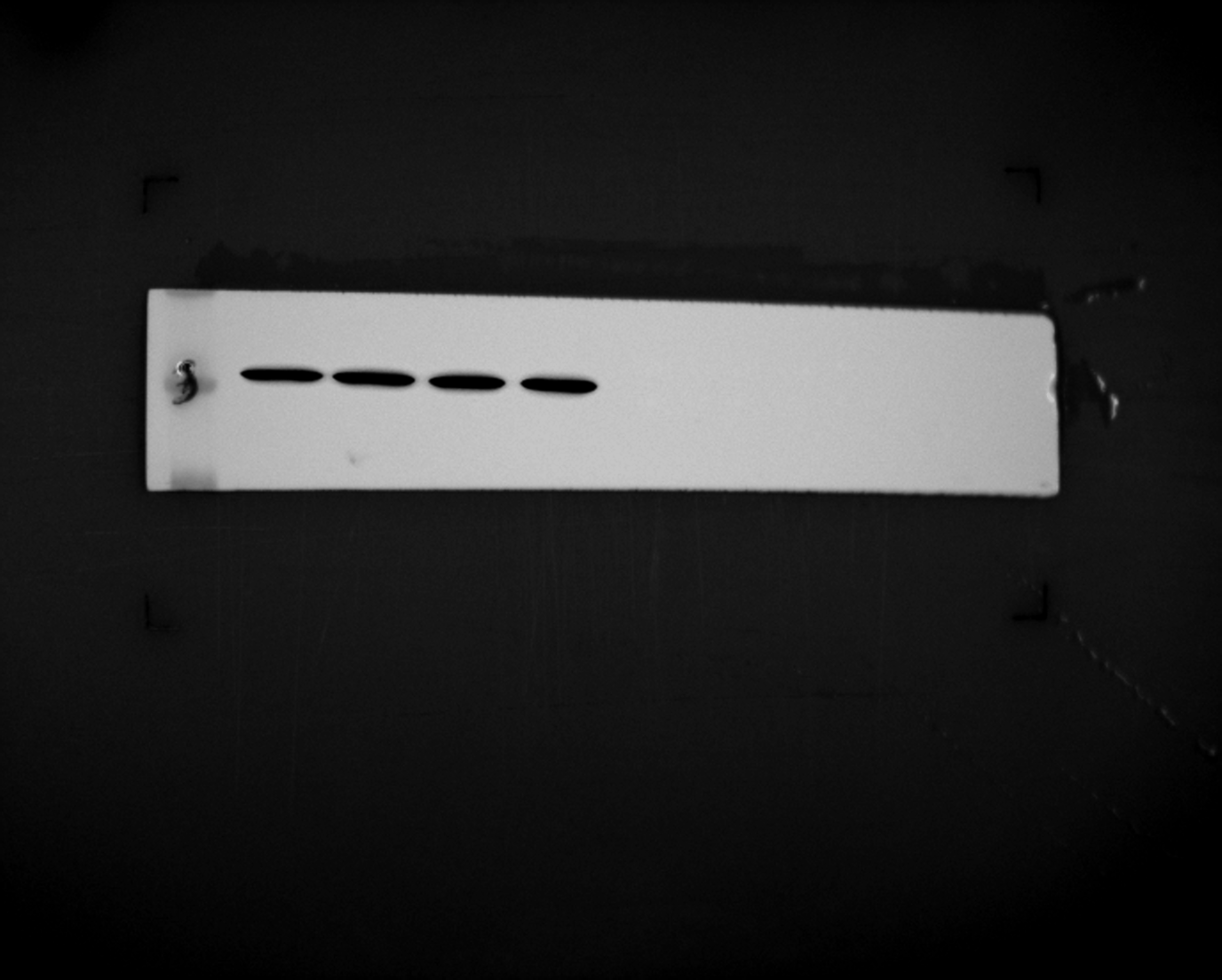

Supplement: Supplemental Material [file KBIE_A_2062106_SM2482.zip › supplementary/Fig5A_GAPDH.tif]

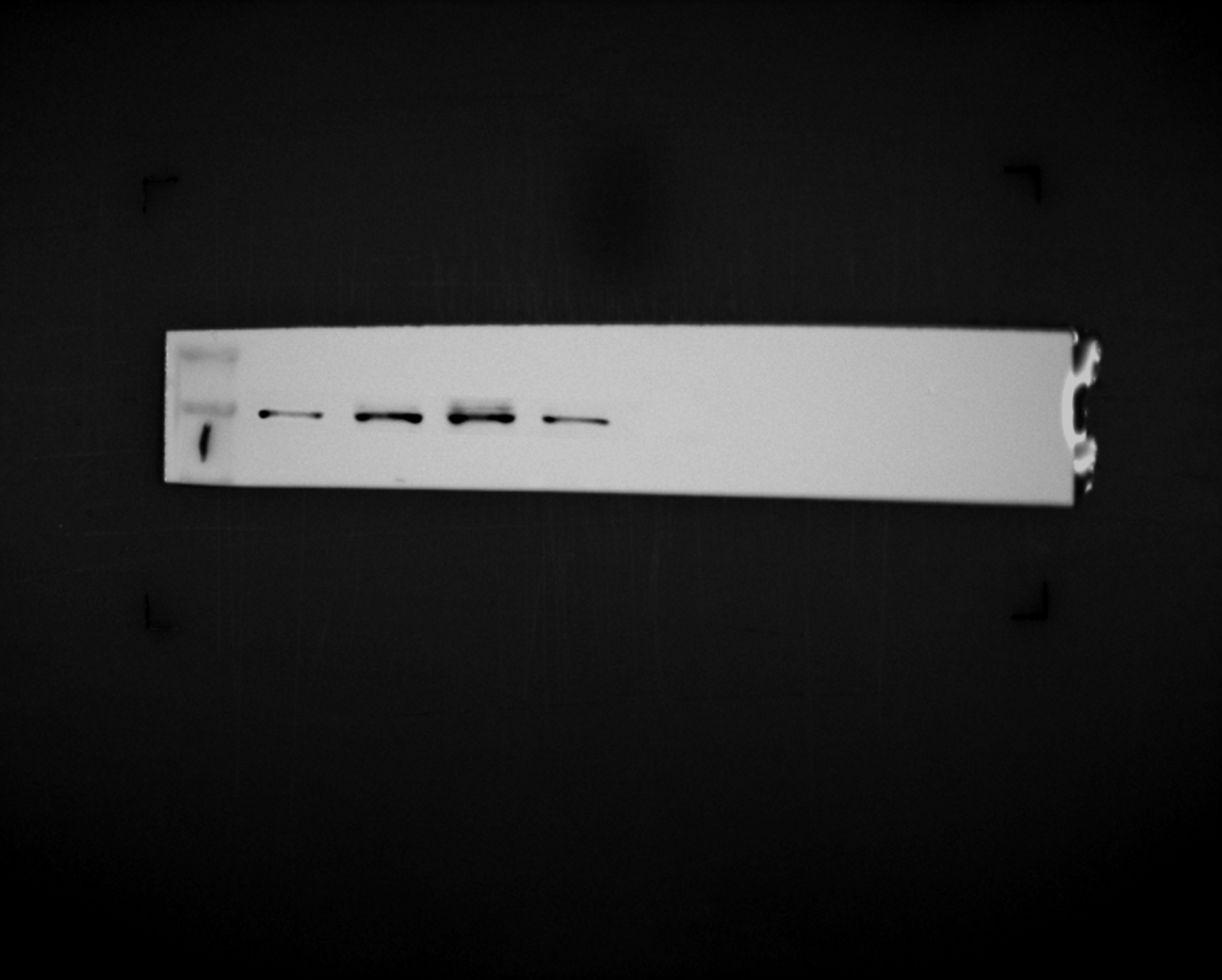

Supplement: Supplemental Material [file KBIE_A_2062106_SM2482.zip › supplementary/Fig5A_pAKT.tif]

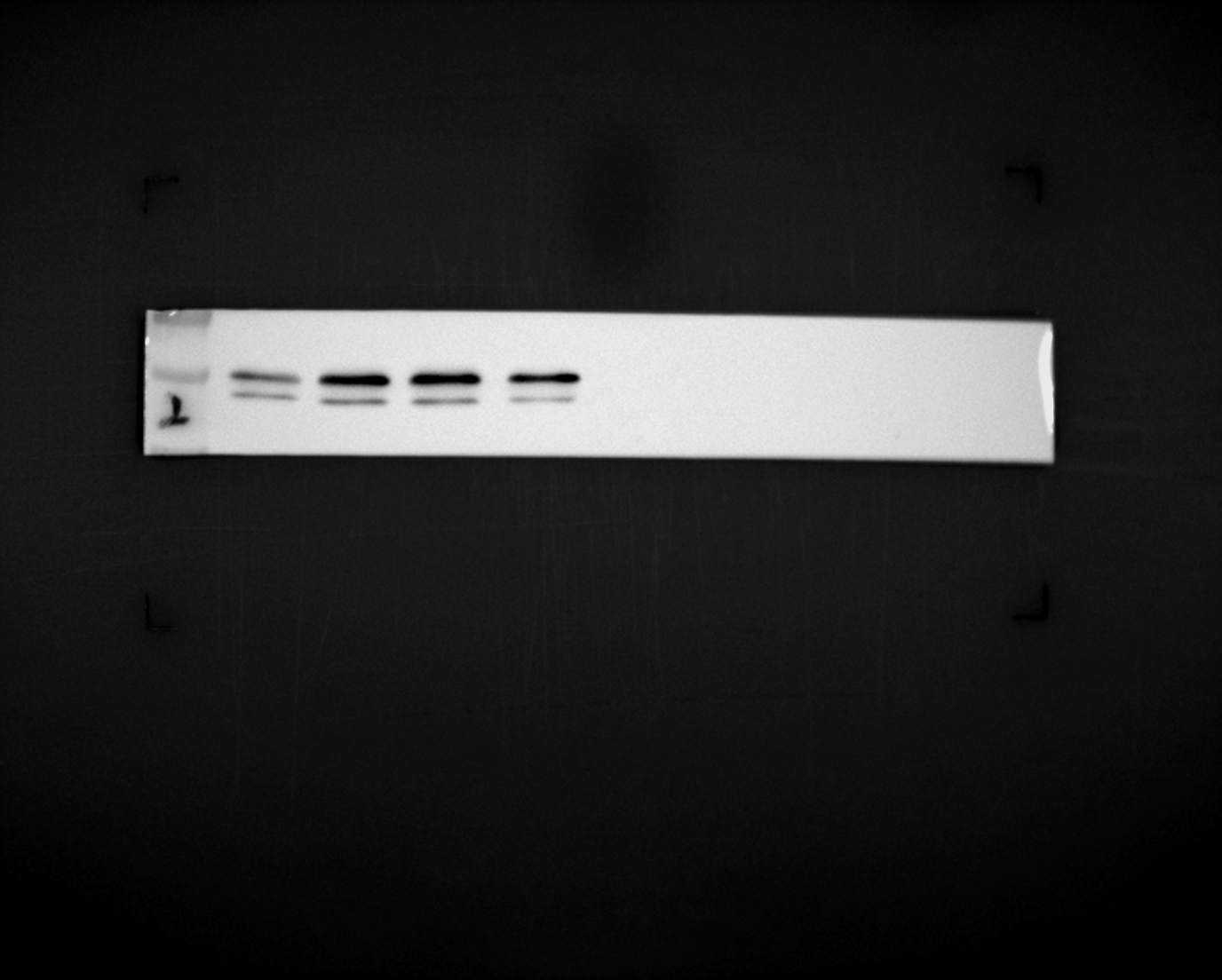

Supplement: Supplemental Material [file KBIE_A_2062106_SM2482.zip › supplementary/Fig5A_psmad.tif]

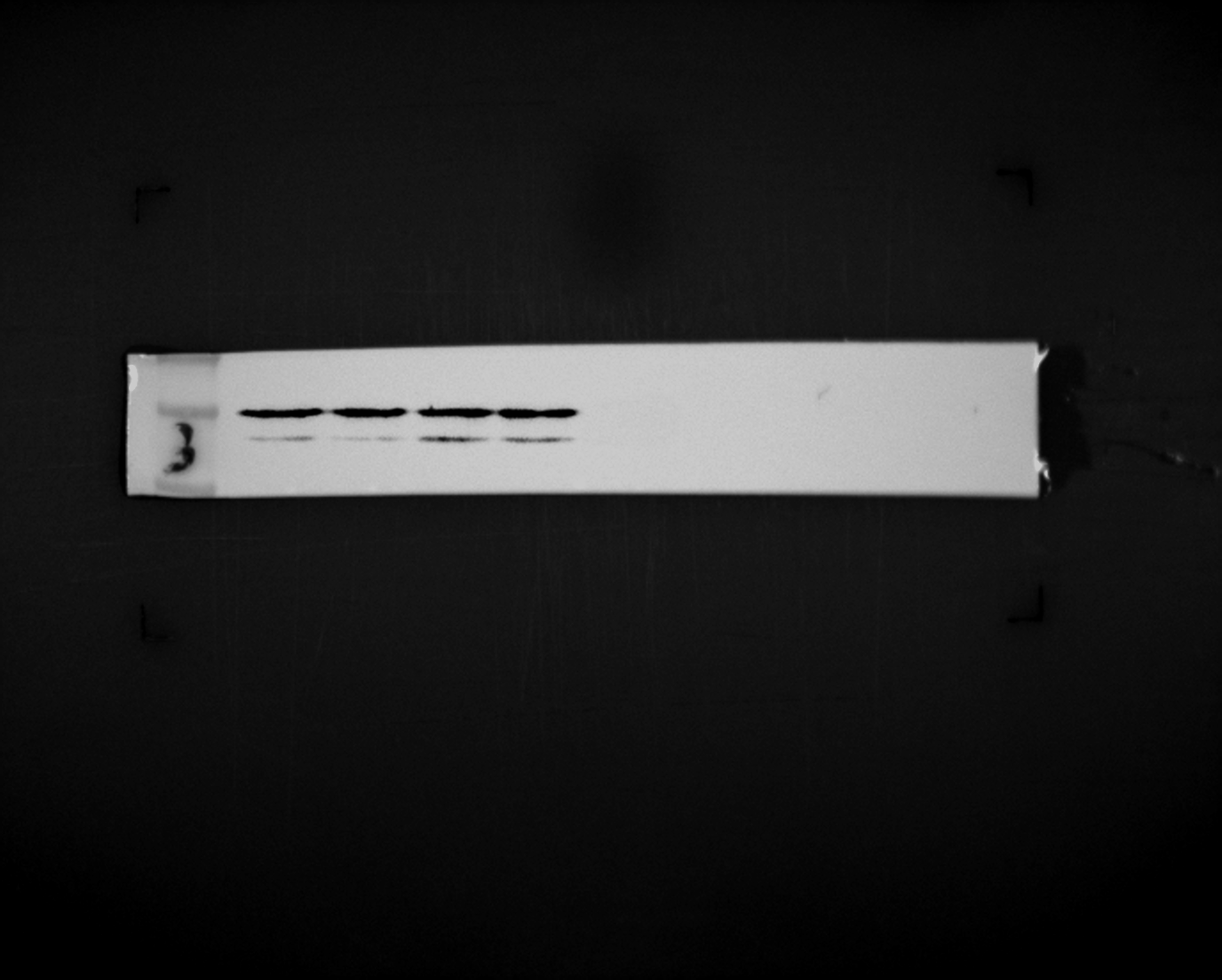

Supplement: Supplemental Material [file KBIE_A_2062106_SM2482.zip › supplementary/Fig5A_smad.tif]

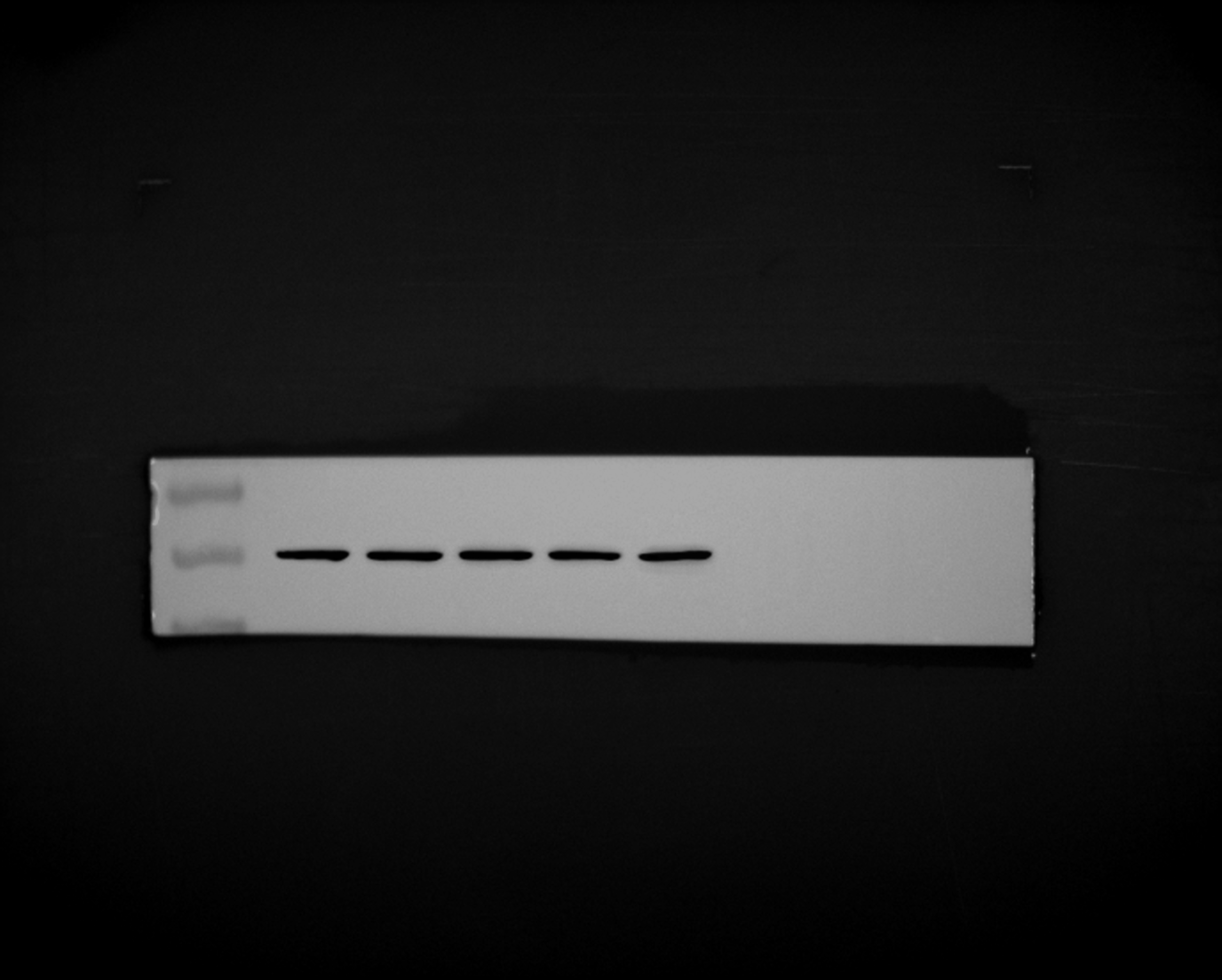

Supplement: Supplemental Material [file KBIE_A_2062106_SM2482.zip › supplementary/Fig5C_AKT.tif]

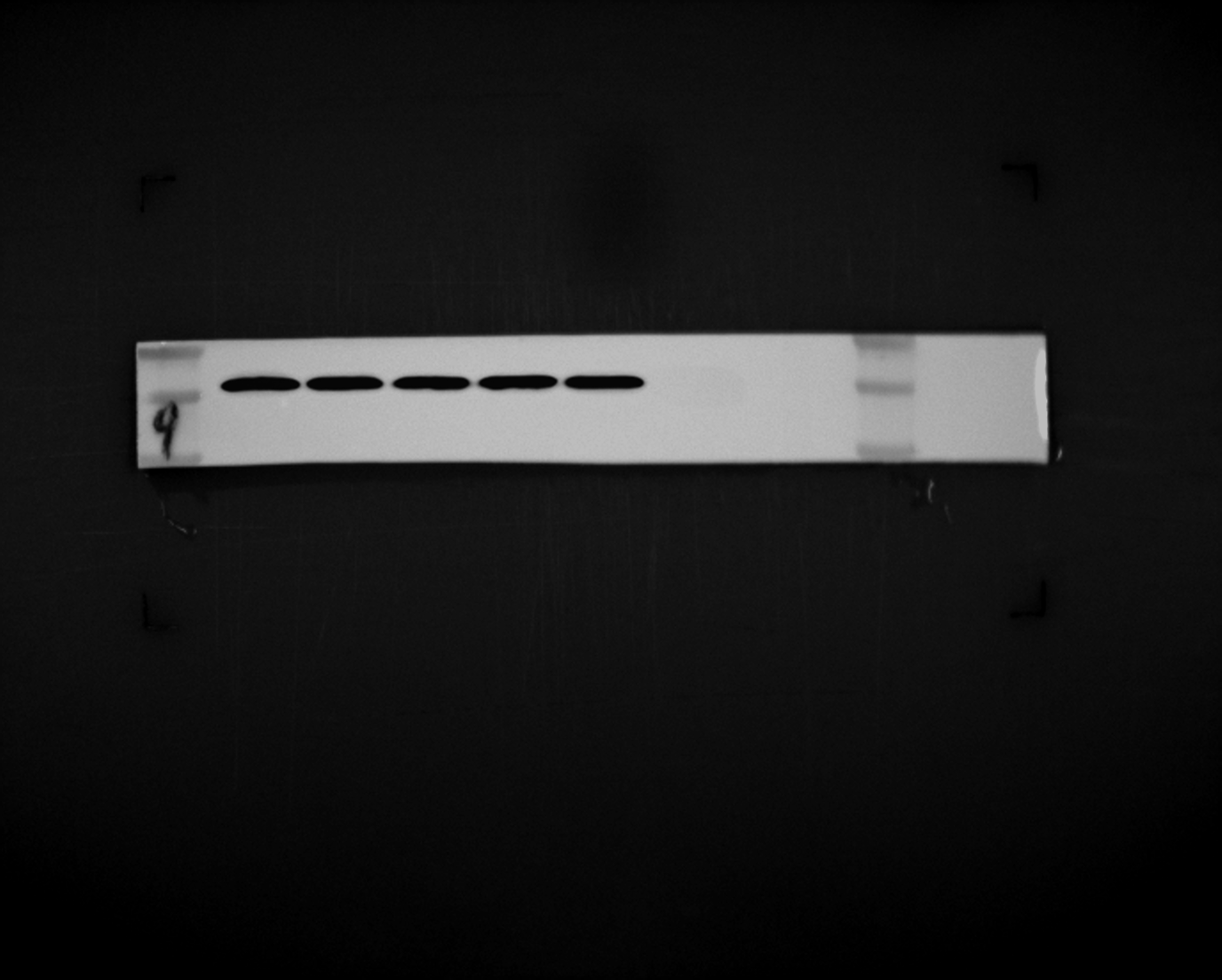

Supplement: Supplemental Material [file KBIE_A_2062106_SM2482.zip › supplementary/Fig5C_GAPDH.tif]

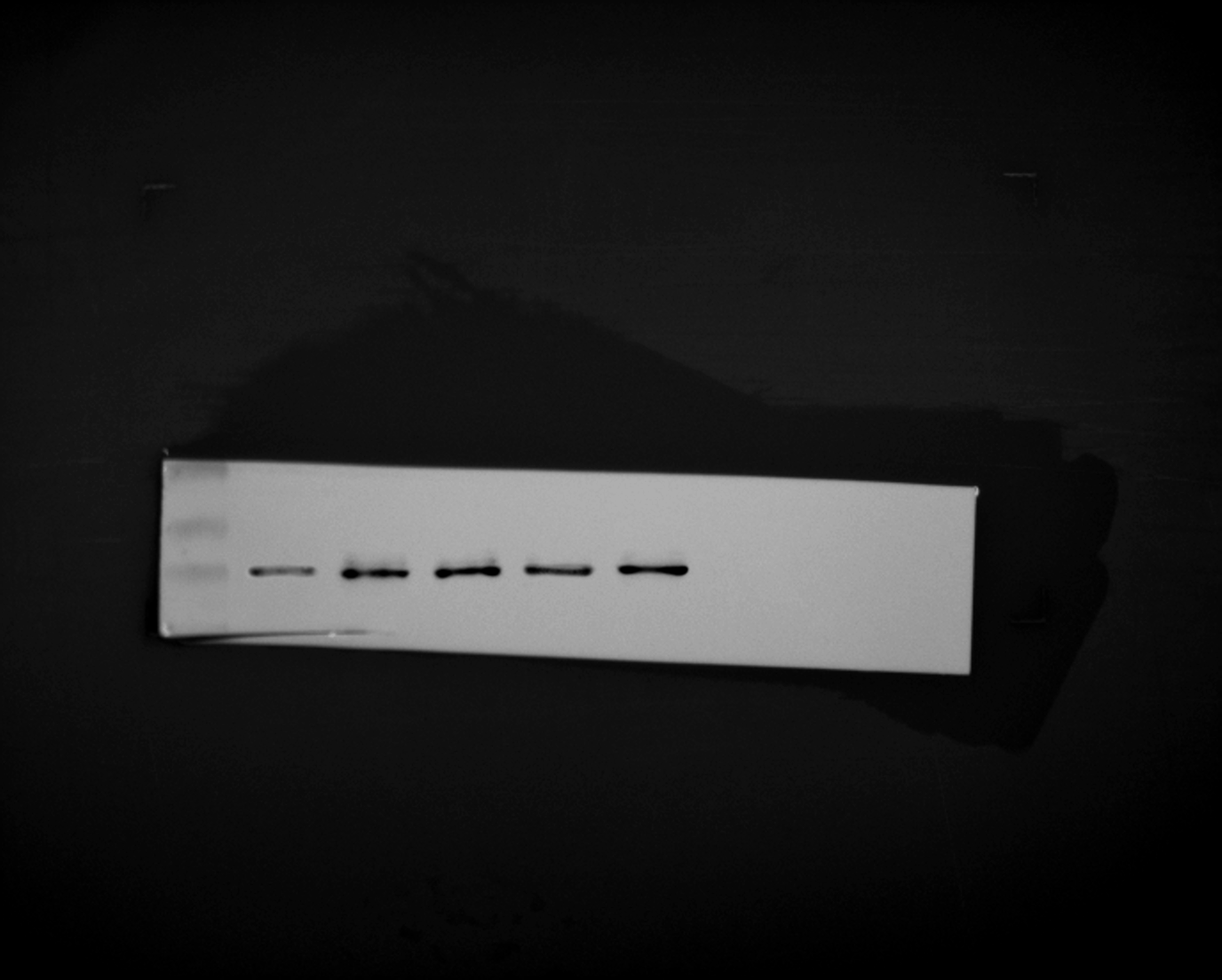

Supplement: Supplemental Material [file KBIE_A_2062106_SM2482.zip › supplementary/Fig5C_pAKT.tif]

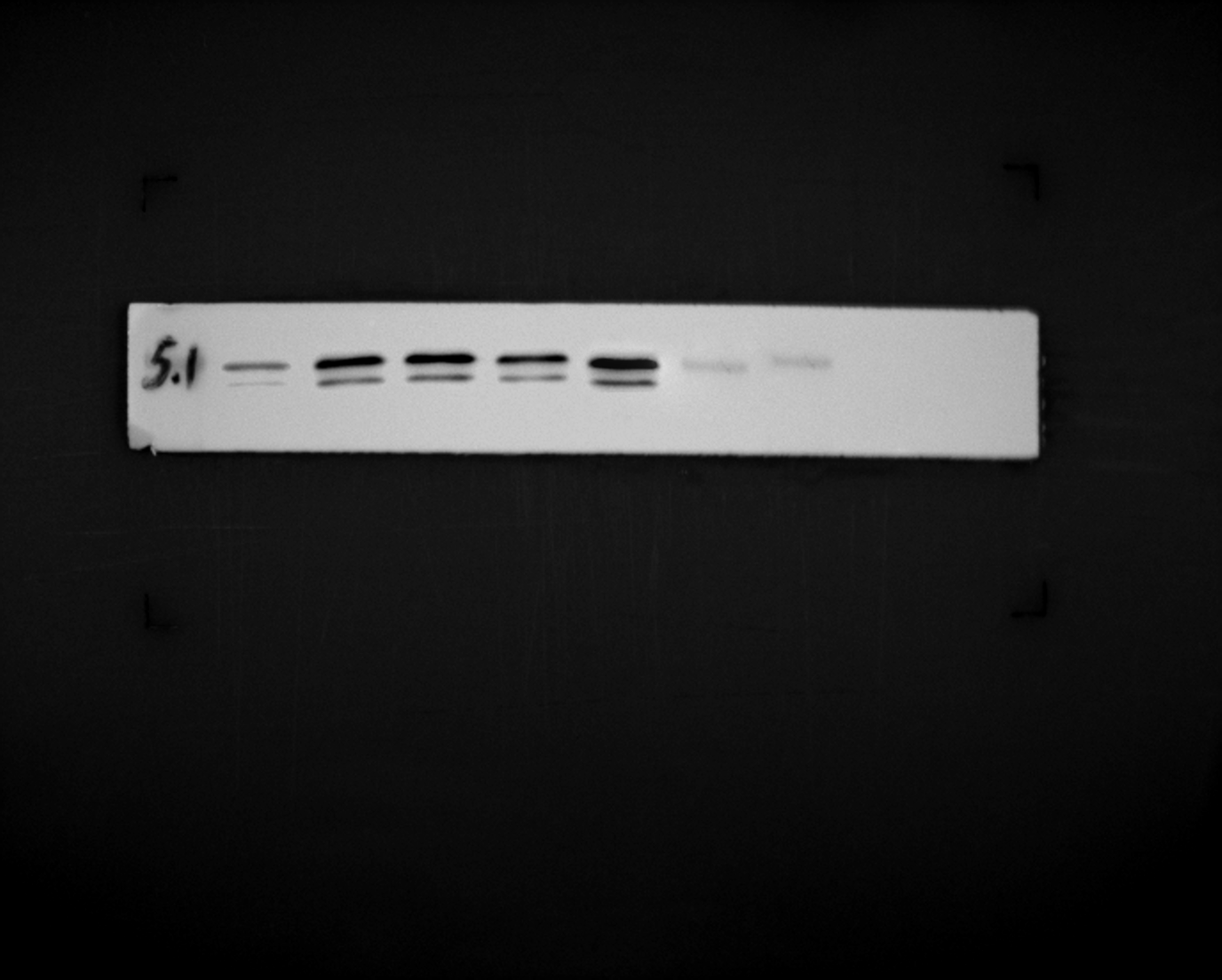

Supplement: Supplemental Material [file KBIE_A_2062106_SM2482.zip › supplementary/Fig5C_psmad23.tif]

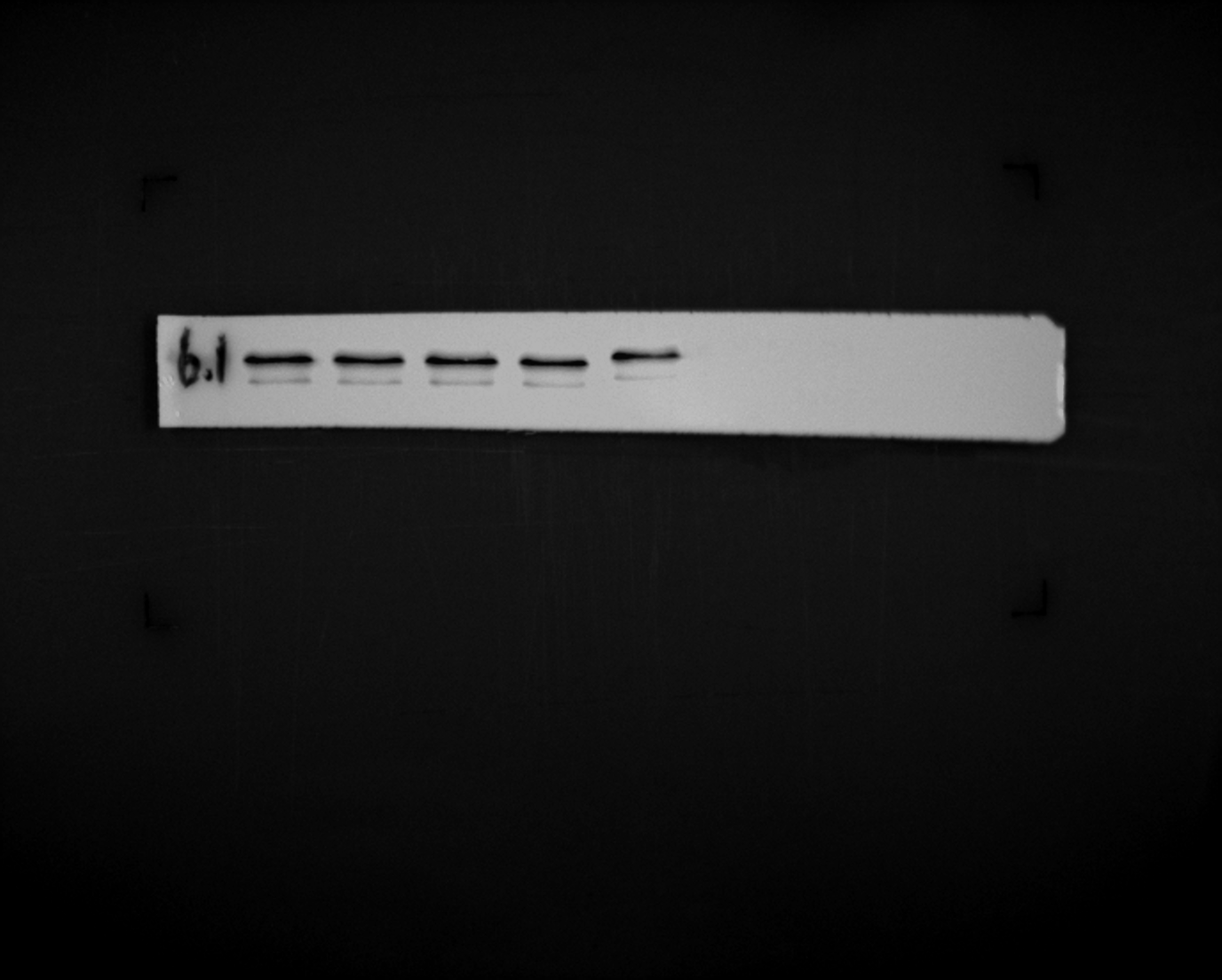

Supplement: Supplemental Material [file KBIE_A_2062106_SM2482.zip › supplementary/Fig5C_smad23.tif]

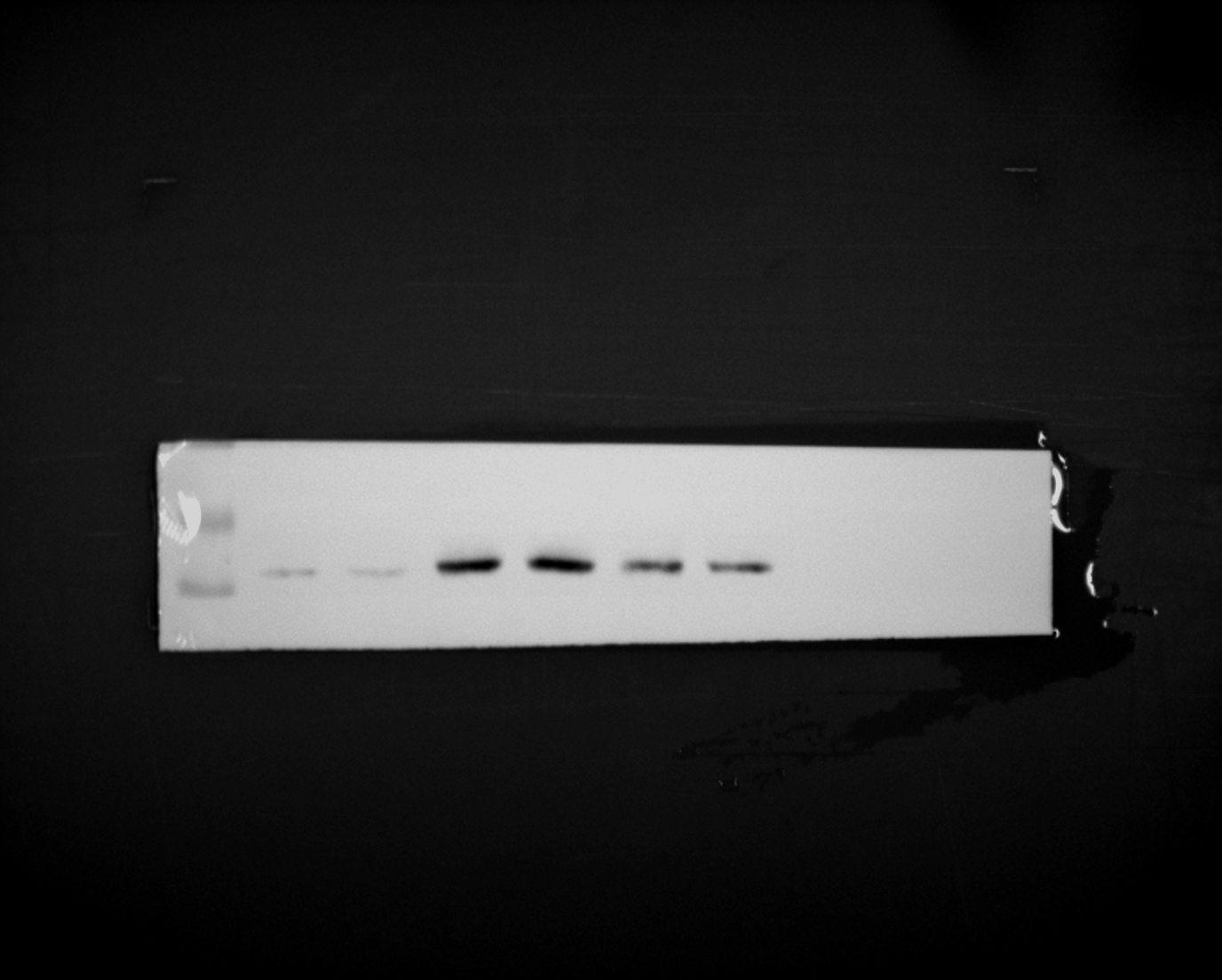

Supplement: Supplemental Material [file KBIE_A_2062106_SM2482.zip › supplementary/Fig6E_CCL20.tif]

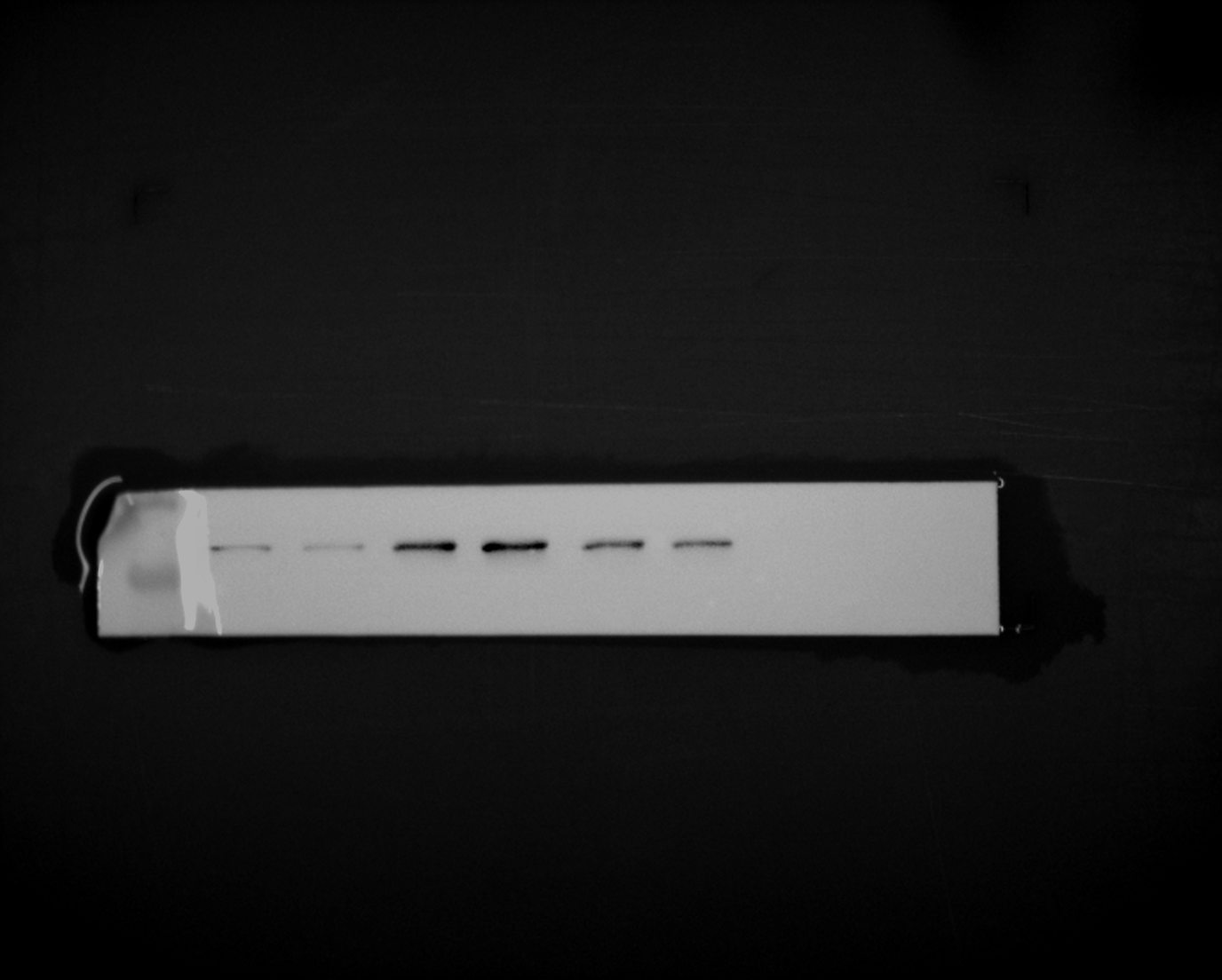

Supplement: Supplemental Material [file KBIE_A_2062106_SM2482.zip › supplementary/Fig6E_CCR6.tif]

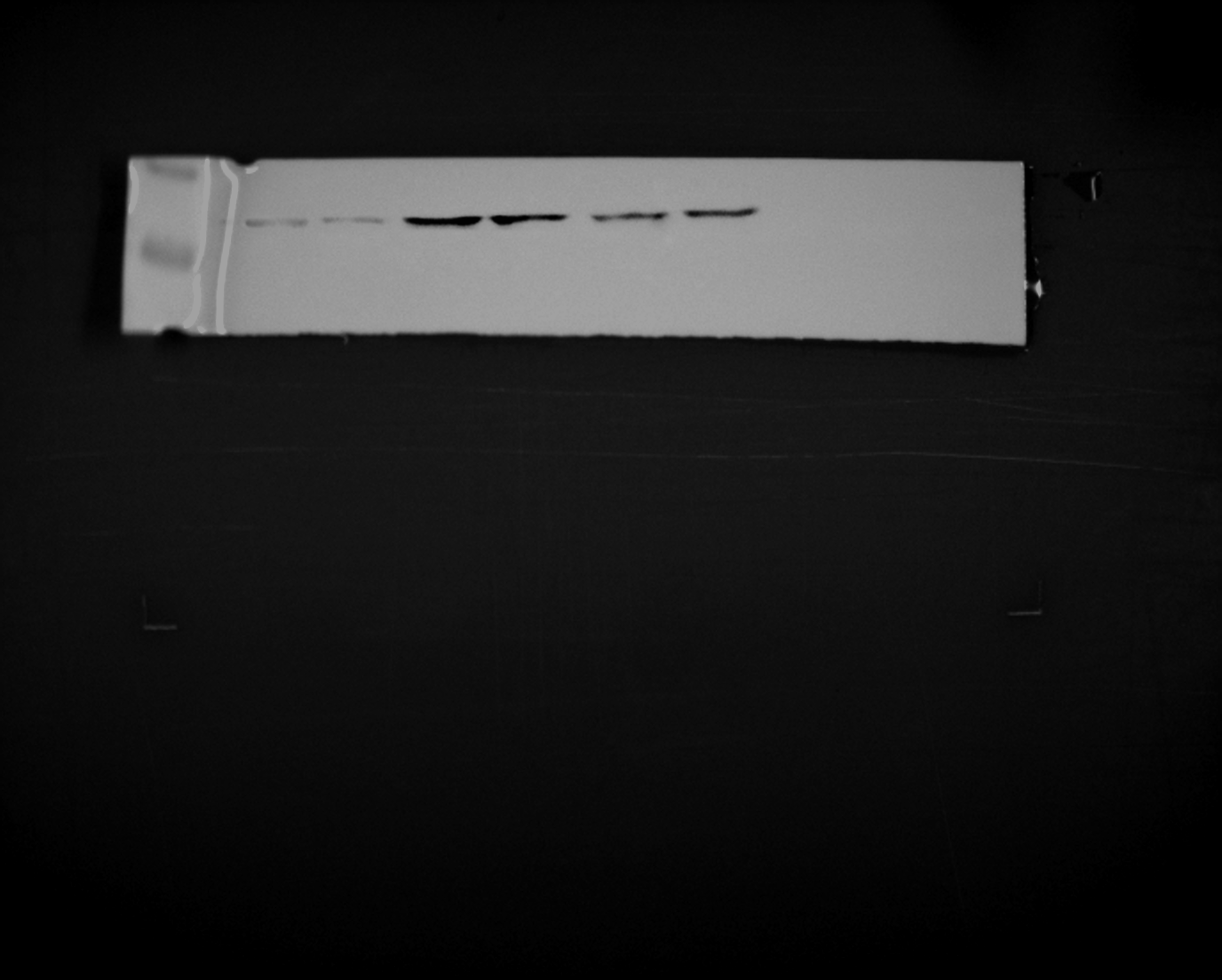

Supplement: Supplemental Material [file KBIE_A_2062106_SM2482.zip › supplementary/Fig6E_CollagenI.tif]

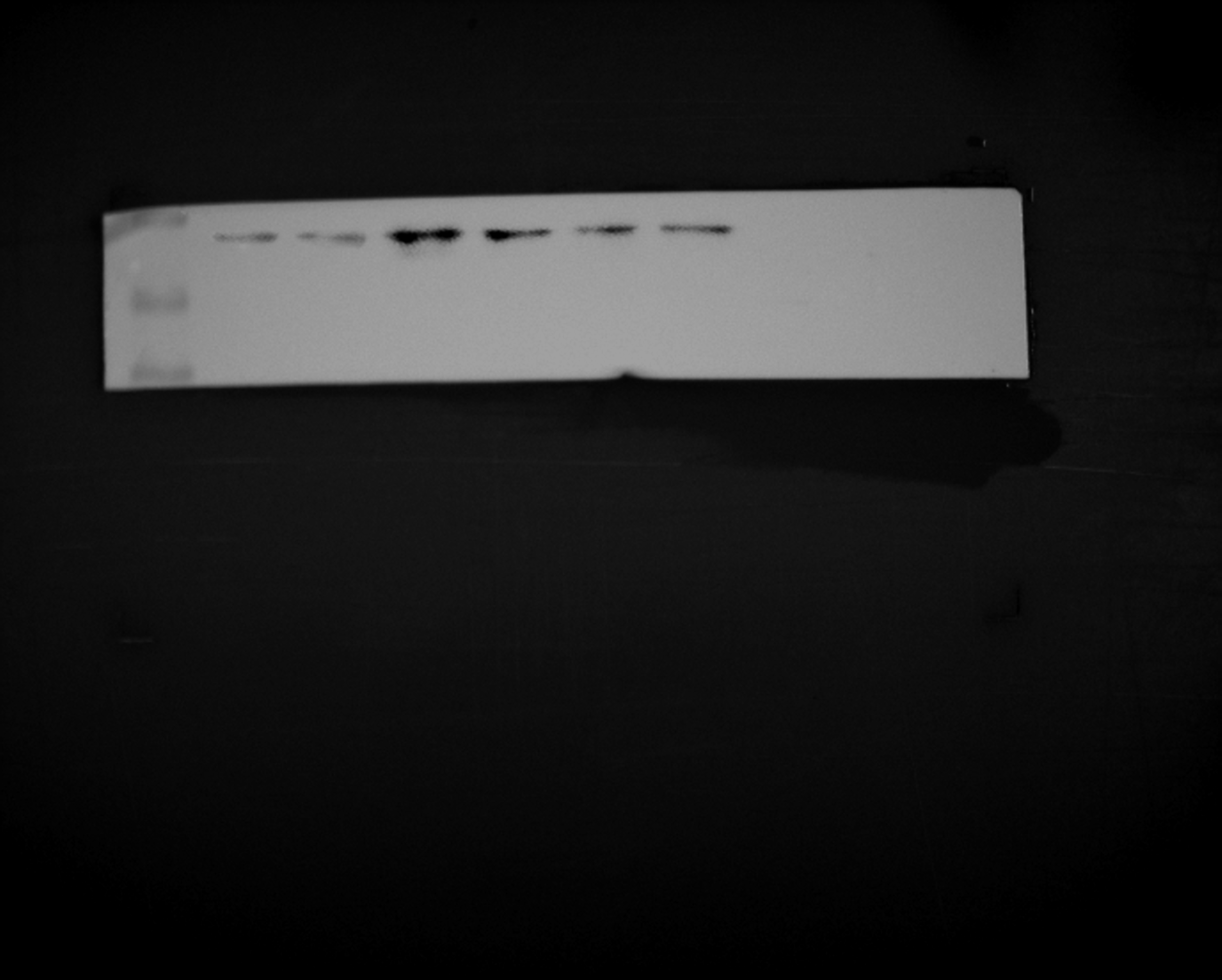

Supplement: Supplemental Material [file KBIE_A_2062106_SM2482.zip › supplementary/Fig6E_FN.tif]

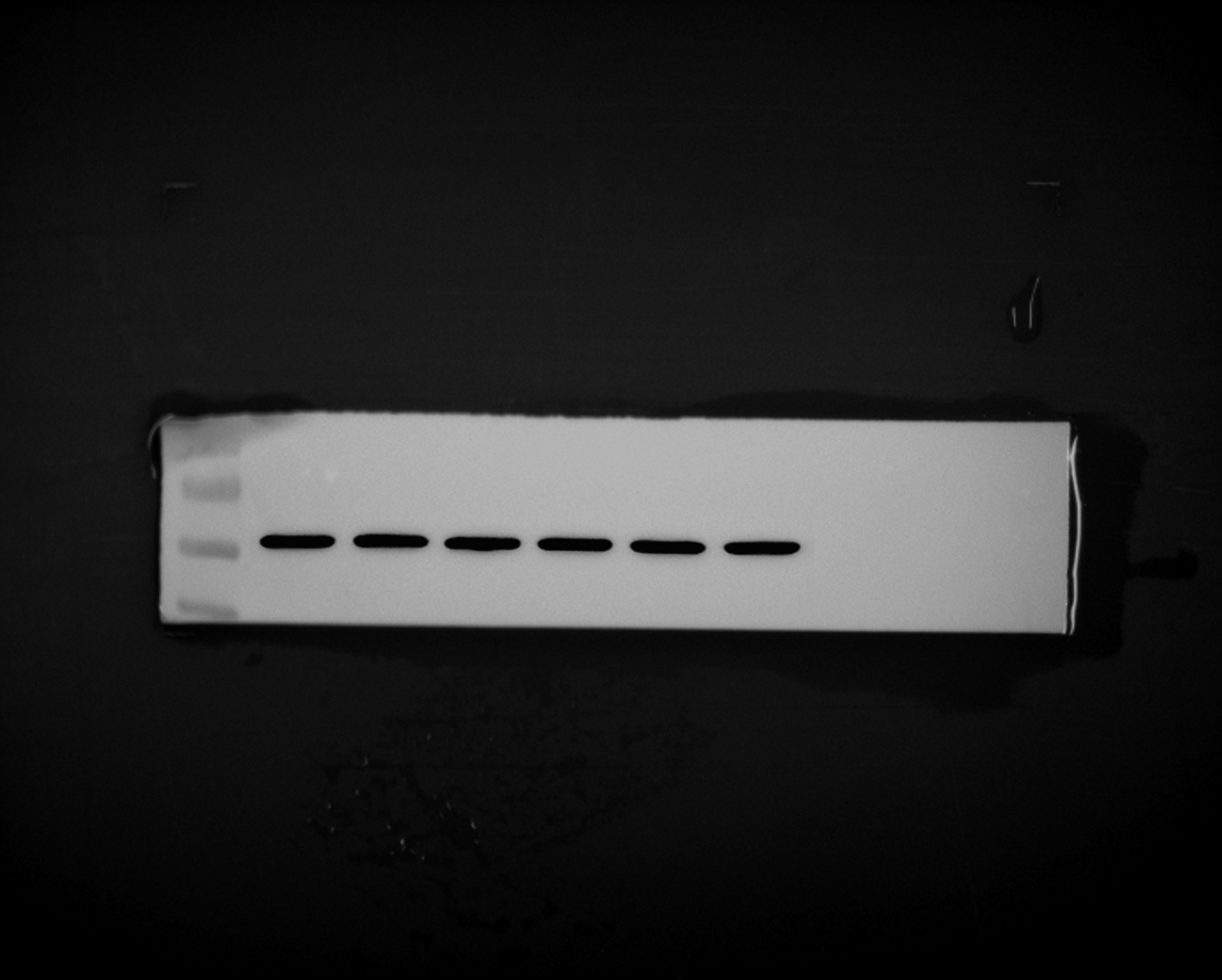

Supplement: Supplemental Material [file KBIE_A_2062106_SM2482.zip › supplementary/Fig6E_GAPDH.tif]

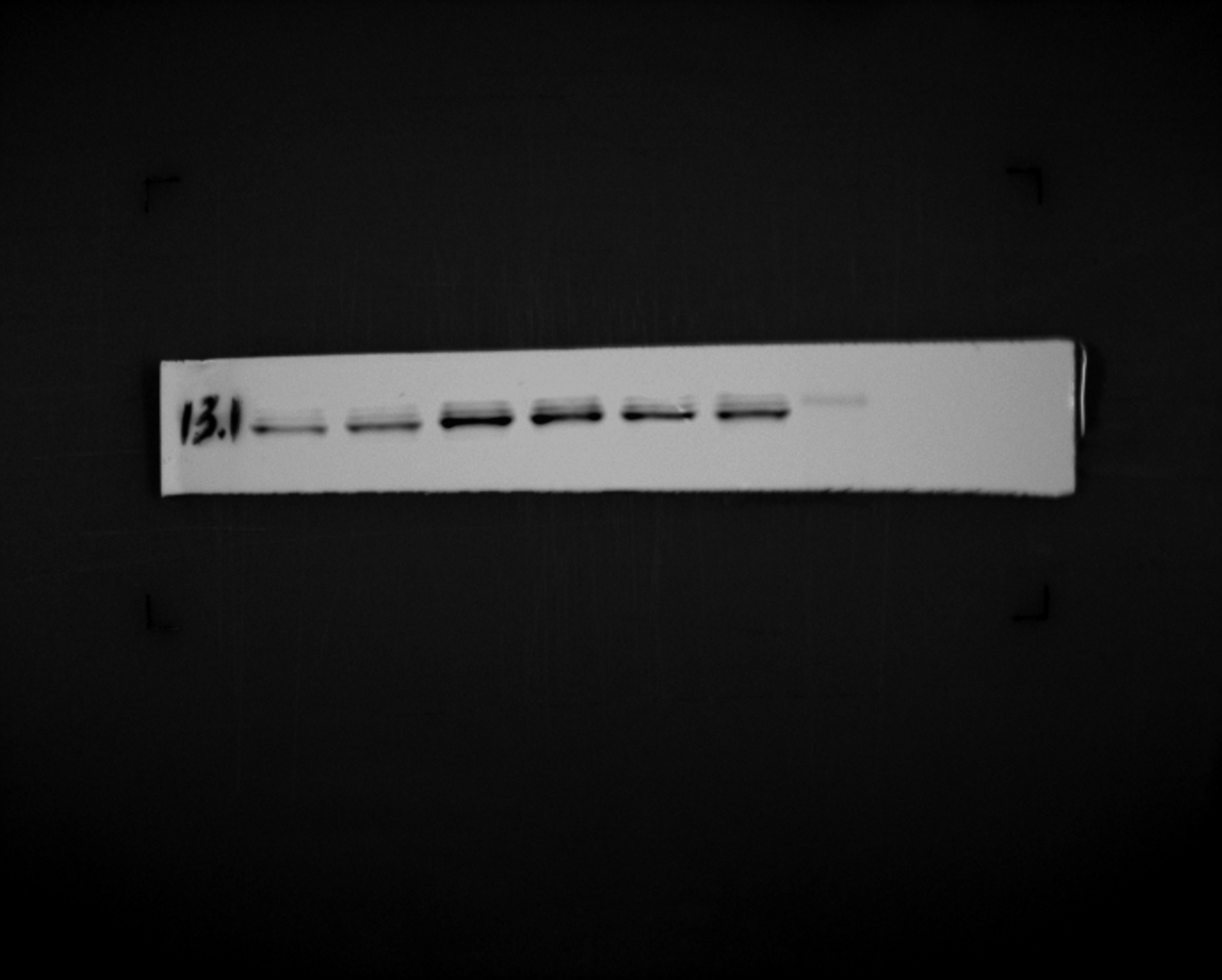

Supplement: Supplemental Material [file KBIE_A_2062106_SM2482.zip › supplementary/Fig6E_TGF_1.tif]

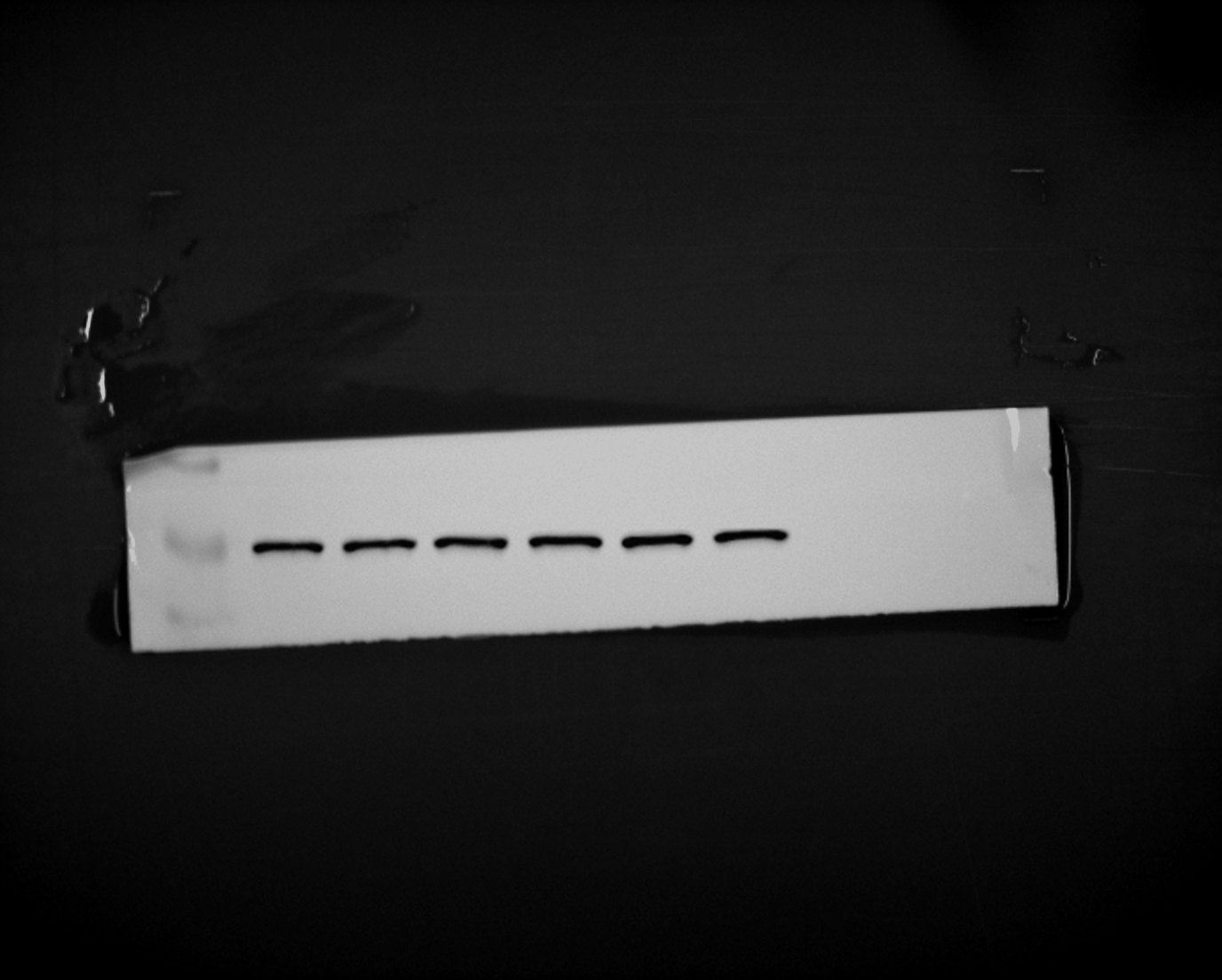

Supplement: Supplemental Material [file KBIE_A_2062106_SM2482.zip › supplementary/Fig6I_AKT.tif]

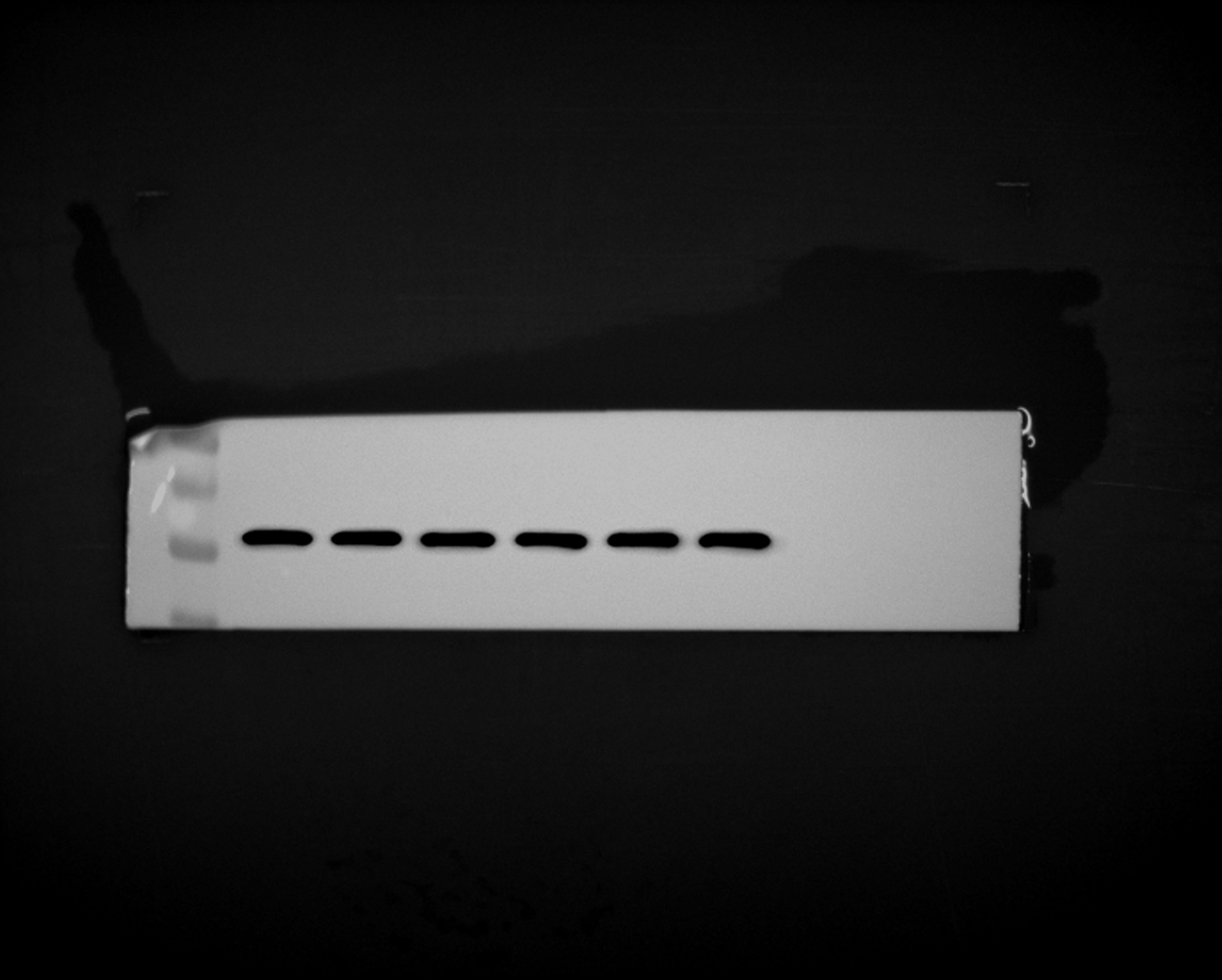

Supplement: Supplemental Material [file KBIE_A_2062106_SM2482.zip › supplementary/Fig6I_GAPDH.tif]

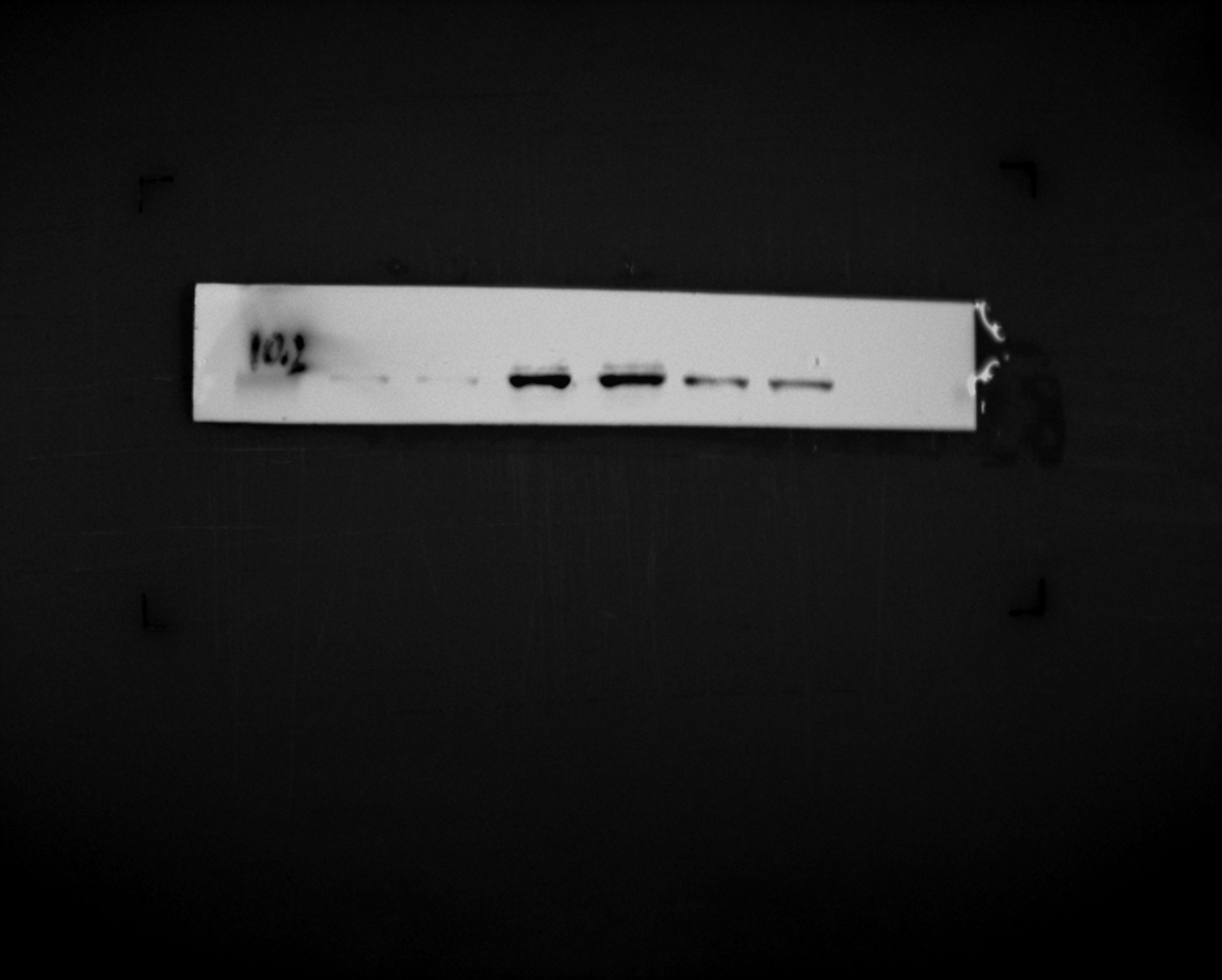

Supplement: Supplemental Material [file KBIE_A_2062106_SM2482.zip › supplementary/Fig6I_pAKT.tif]

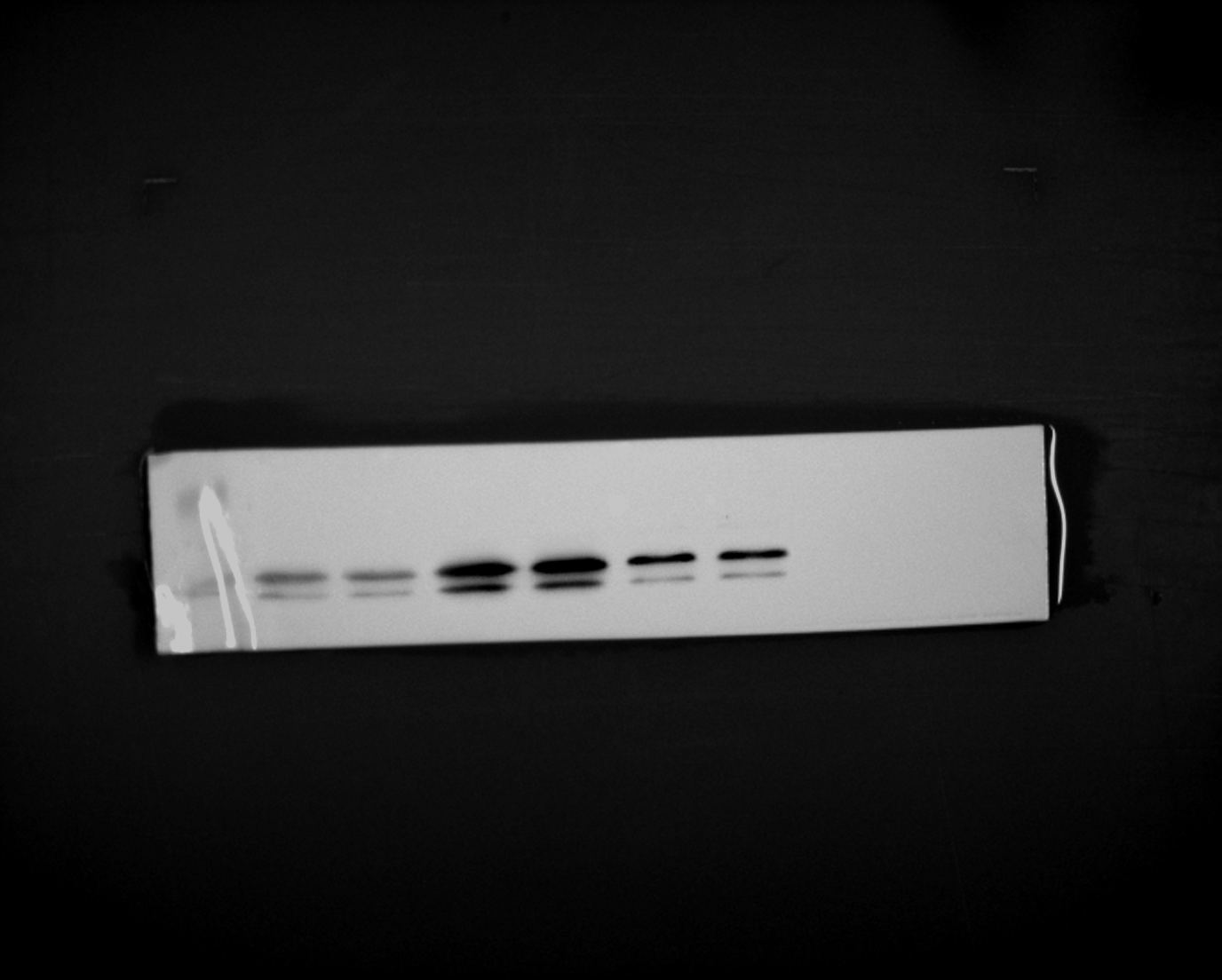

Supplement: Supplemental Material [file KBIE_A_2062106_SM2482.zip › supplementary/Fig6I_psmad23.tif]

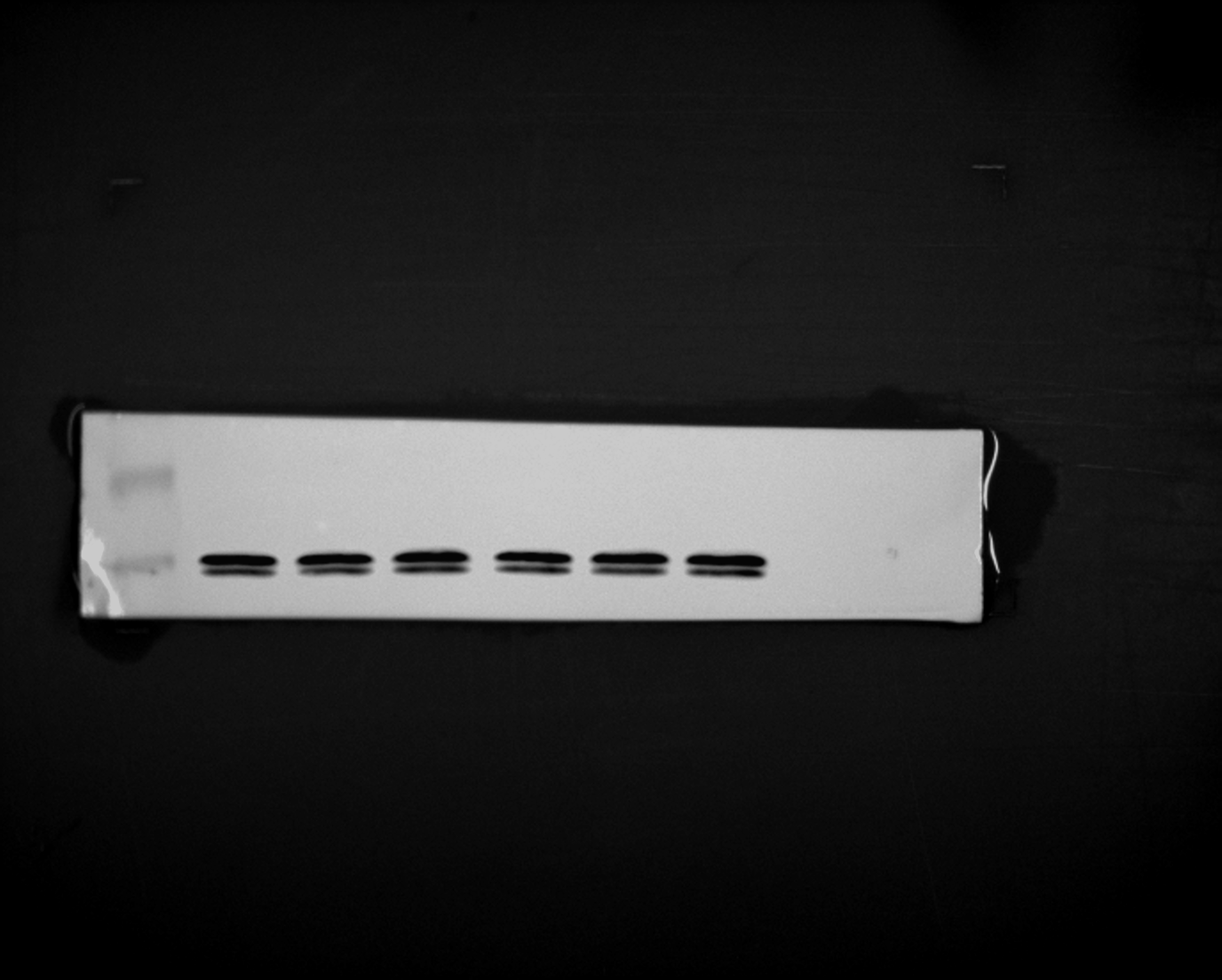

Supplement: Supplemental Material [file KBIE_A_2062106_SM2482.zip › supplementary/Fig6I_smad23.tif]
